# Global characterization of interferon regulatory factor (IRF) genes in vertebrates: Glimpse of the diversification in evolution

**DOI:** 10.1186/1471-2172-11-22

**Published:** 2010-05-05

**Authors:** Bei Huang, Zhi T Qi, Zhen Xu, Pin Nie

**Affiliations:** 1 State Key Laboratory of Freshwater Ecology and Biotechnology, Institute of Hydrobiology, Chinese Academy of Sciences, Wuhan, Hubei Province, 430072, China; 2 Graduate School of Chinese Academy of Sciences, Beijing 10049, China

## Abstract

**Background:**

Interferon regulatory factors (IRFs), which can be identified based on a unique helix-turn-helix DNA-binding domain (DBD) are a large family of transcription factors involved in host immune response, haemotopoietic differentiation and immunomodulation. Despite the identification of ten IRF family members in mammals, and some recent effort to identify these members in fish, relatively little is known in the composition of these members in other classes of vertebrates, and the evolution and probably the origin of the IRF family have not been investigated in vertebrates.

**Results:**

Genome data mining has been performed to identify any possible IRF family members in human, mouse, dog, chicken, anole lizard, frog, and some teleost fish, mainly zebrafish and stickleback, and also in non-vertebrate deuterostomes including the hemichordate, cephalochordate, urochordate and echinoderm. In vertebrates, all ten IRF family members, i.e. IRF-1 to IRF-10 were identified, with two genes of IRF-4 and IRF-6 identified in fish and frog, respectively, except that in zebrafish exist three IRF-4 genes. Surprisingly, an additional member in the IRF family, IRF-11 was found in teleost fish. A range of two to ten IRF-like genes were detected in the non-vertebrate deuterostomes, and they had little similarity to those IRF family members in vertebrates as revealed in genomic structure and in phylogenetic analysis. However, the ten IRF family members, IRF-1 to IRF-10 showed certain degrees of conservation in terms of genomic structure and gene synteny. In particular, IRF-1, IRF-2, IRF-6, IRF-8 are quite conserved in their genomic structure in all vertebrates, and to a less degree, some IRF family members, such as IRF-5 and IRF-9 are comparable in the structure. Synteny analysis revealed that the gene loci for the ten IRF family members in vertebrates were also quite conservative, but in zebrafish conserved genes were distributed in a much longer distance in chromosomes. Furthermore, all ten different members are clustered in respectively different clades; but the IRF-11 was clustered with one in sea urchin.

**Conclusions:**

In vertebrates, the ten well-characterized IRF family members shared a relatively high degree of similarity in genomic structure and syntenic gene arrangement, implying that they might have been evolved in a similar pattern and with similar selective pressure in different classes of vertebrates. Genome and/or gene duplication, and probably gene shuffling or gene loss might have occurred during the evolution of these IRF family members, but arrangement of chromosome or its segment might have taken place in zebrafish. However, the ten IRF family members in vertebrates and those IRF-like genes in non-vertebrate deuterostomes were quite different in those analyzed characters, as they might have undergone different patterns of evolution.

## Background

Interferon regulatory factors (IRFs) were identified originally as transcription factors in the regulation of interferon expression [1]. Over the last a few decades, these factors have been the focus of many immunological and medical studies [2,3], and it has been shown that they have diversified functions in immune responses, haematopoietic differentiation and immune modulation [4-6]. These transcription factors posses a unique 'tryptophan cluster' DNA-binding domain (DBD) [7], which is responsible for binding to the IFN-regulatory factor element present in the IFN-β promoter [8]. Since the identification of the first IRF, IRF-1, as a protein binding to the *cis*-acting DNA elements of the IFN-β gene, a total of ten members has been identified in vertebrates with functions from activators (e.g., IRF-1, IRF-3, IRF-5, IRF-9 and IRF-10), to repressors (e.g., IRF-8); and some of them (e.g., IRF-2, IRF-4, and IRF-7) also exert the two functions [3,4,9,10]. Previous research has largely focused on the function of individual IRFs in mammals [8,11,12]. However, the composition and the function of these IRF family members are less investigated in other classes of vertebrates [13]. In fish, a few members, such as IRF-1, IRF-2 and IRF-7 have been cloned in some aquacultured species [14-18], and recently a total of eleven IRF family members has been identified in zebrafish *Dario rerio *[19].

In an attempt to clearly delineate the evolution of IRF family lineage, we analyzed the draft genomes of human (*Homo sapiens*), mouse (*Mus musculus*), dog (*Canis familiaris*), chicken (*Gallus gallus*), anole lizard (*Anolis carolinesis*), frog (*Xenopus tropicalis*), zebrafish (*Dario rerio*), fugu (*Takifugu rubripes*), stickleback (*Gasterosteus aculeatus*), medaka (*Oryzias latipes*) to systematically identify all IRF members in each species. The searched results were further compared with literature and the location of each member in the IRF family was mapped physically on chromosomes in human, mouse, dog, chicken, anole lizard, frog and zebrafish representing classes of mammal, avian, reptile, amphibian and piscine. In addition, any possible IRF-like genes were identified in some non-vertebrate deuterostomes, including the hemichordate, acorn worm *Saccoglossus kowalevskii*, the echinoderm, sea urchin *Strongylocentrotus purpuratus*, the cephalochordate, lancelet (amphioxus) *Branchiostoma floridae *and the urochordate, sea squirt *Ciona intestinalis*. The conservation in the genome organization of individual IRF family members and the synteny in their gene loci were analyzed. The possible evolutionary mechanisms in the origination of IRF family members were then discussed.

## Results

A total of ten members in the IRF family, i.e., IRF- 1 to 10 have been identified in vertebrates, with another member named as IRF-11 in teleost fish, as also in another study [19]. All these members, despite their diversified functions, share a conserved motif of a five tryptophan pentad repeat, with three of them, W-x-[DNH]-x(5)-[LIVF]-x-[IV]-P-W-x-H-x(9,10)-[DE]-x(2)-[LIVF]-F-[KRQ]-x-[WR]-A, contacting with DNA sequence and recognizing the AANNGAAA sequence [7]. In mammals, IRF-10 was not found in human and mouse. In zebrafish, stickleback, frog, anole lizard, chicken and dog, all ten members were detected. However in chicken, IRF-3 and IRF-9 were not found. In addition, any possible IRF-like genes were identified in non-vertebrate deuterostomes, with the finding of three IRF genes in hemichordate, the acorn worm *Saccoglossus kowalevskii*, two in echinoderm, the sea urchin *Strongylocentrotus purpuratus*, ten in cephalochordate, the lancelet *Branchiostoma floridae *and nine in urochordate, the sea squirt *Ciona intestinalis*.

### IRF-1 to IRF-11 in vertebrates

#### IRF-1

**Figure 1:**
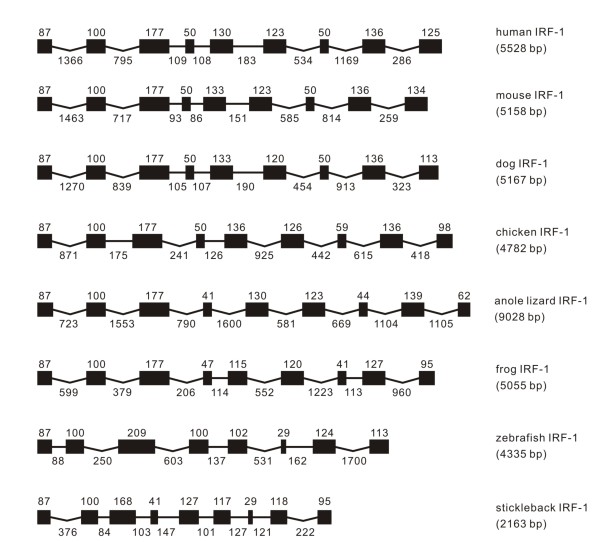
**Genomic structure of IRF-1 in different classes of vertebrates**. The gene names listed on the right are as the same as those in Table 1 and in Additional file 1. Exons are expressed as black boxes, and introns as lines. The size of exons is indicated above the boxes in bp, and the size of introns below the lines also in bp. The length of exons indicated as black boxes and of introns indicated as straight lines is proportional to their bp sizes, but the concave-lines are non-proportional. Human (*Homo sapiens*), mouse (*Mus musculus*) and dog (*Canis familiaris*), chicken (*Gallus gallus*), anole lizard (*Anolis carolinensis*), frog (*Xenopus tropicalis*), and zebrafish (*Dario rerio*) and stickleback (*Gasterosteus aculeatu*s) represent vertebrates in classes of mammal, avian, reptile, amphibian and piscine, as also indicated in Figures 2 to 11.

**Figure 2:**
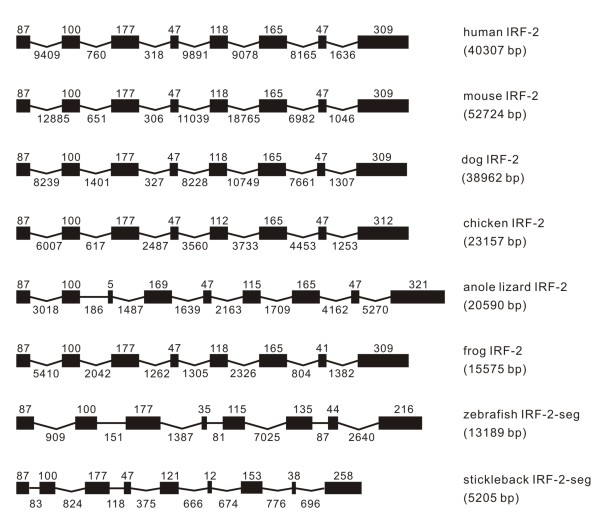
**Genomic structure of IRF-2 in different classes of vertebrates**. The gene names, and exon-intron organizations, as well as the vertebrates are expressed in the same way as appeared in Figure 1, and so for Figures 3 to 10. The gene name followed with -seg indicates that the gene only has a sequence segment in database, as also indicated in Table 1 and in Additional file 1, and also in other figures.

**Figure 3:**
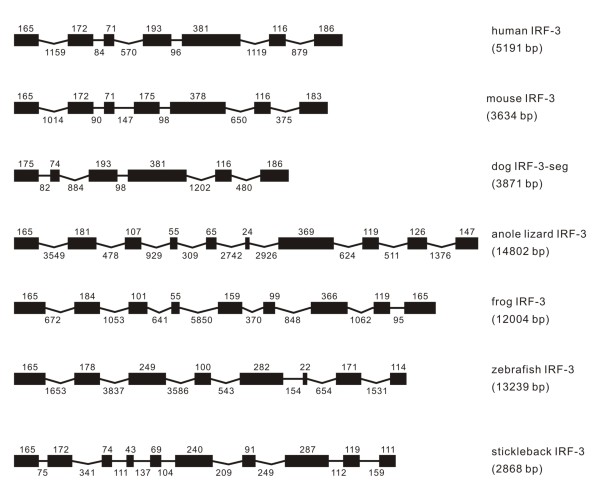
**Genomic structure of IRF-3 in different classes of vertebrates, except that in chicken the IRF-3 was not found**.

**Figure 4:**
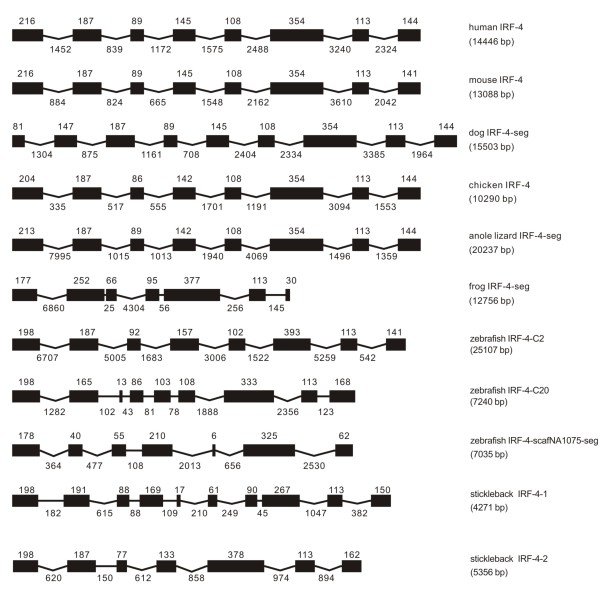
**Genomic structure of IRF-4 in different classes of vertebrates**. Multi-copy genes of IRF-4 were found in teleost fish.

**Figure 5:**
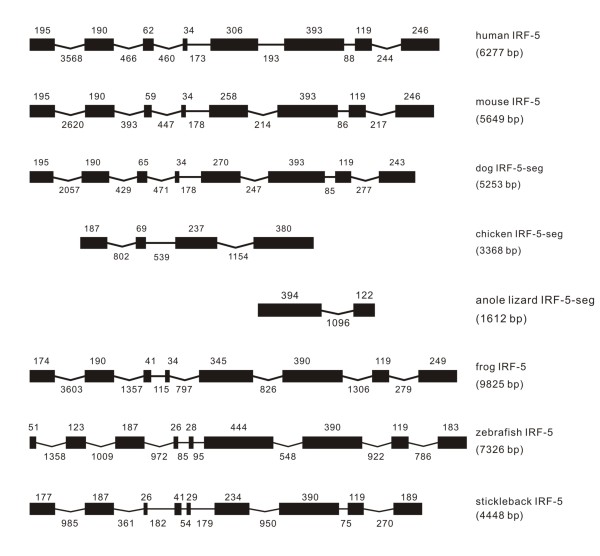
**Genomic structure of IRF-5 in different classes of vertebrates**.

**Figure 6:**
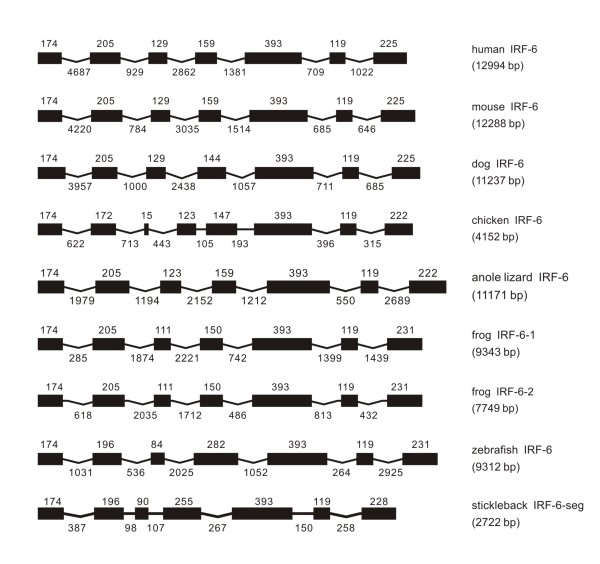
**Genomic structure of IRF-6 in different classes of vertebrates**. Two identical IRF-6 genes were found in frog.

**Figure 7:**
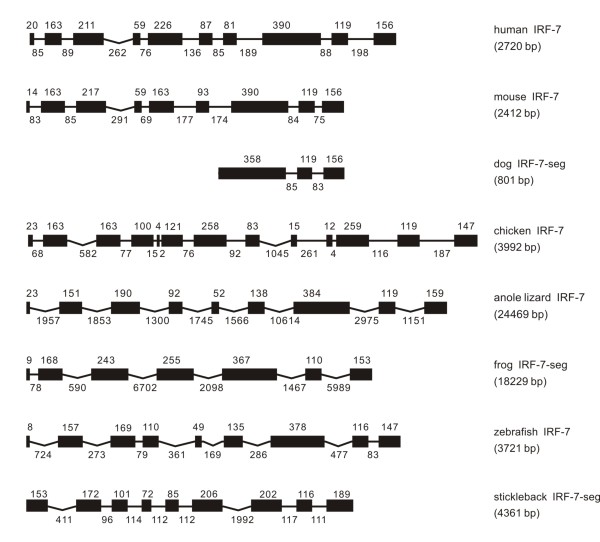
**Genomic structure of IRF-7 in different classes of vertebrates**.

**Figure 8:**
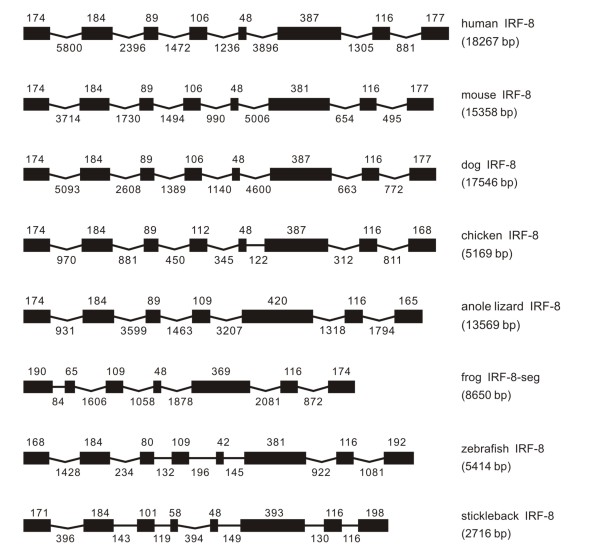
**Genomic structure of IRF-8 in different classes of vertebrates**.

**Figure 9:**
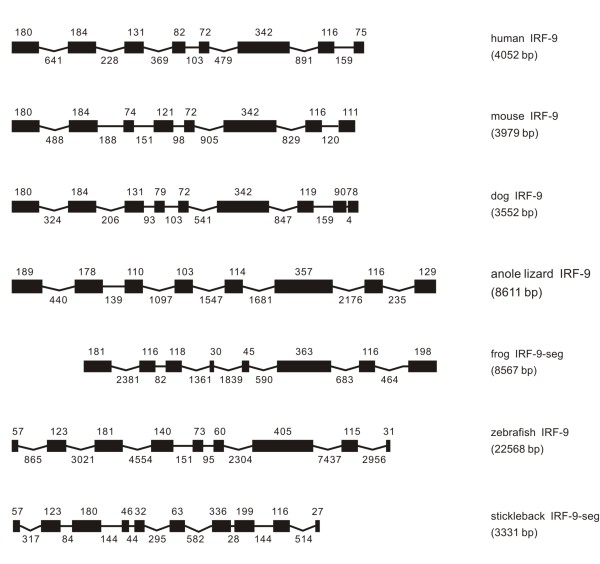
**Genomic structure of IRF-9 in different classes of vertebrates, except that in chicken the IRF-9 was not found**.

**Figure 10:**
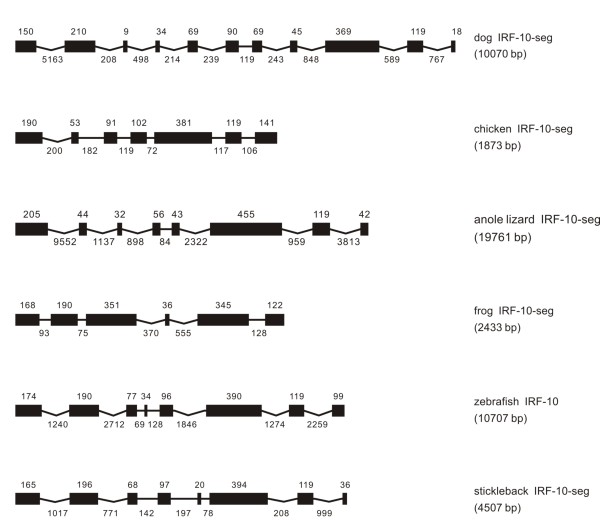
**Genomic structure of IRF-10 in different classes of vertebrates**. IRF-10 was not found in human and mouse

**Figure 11:**
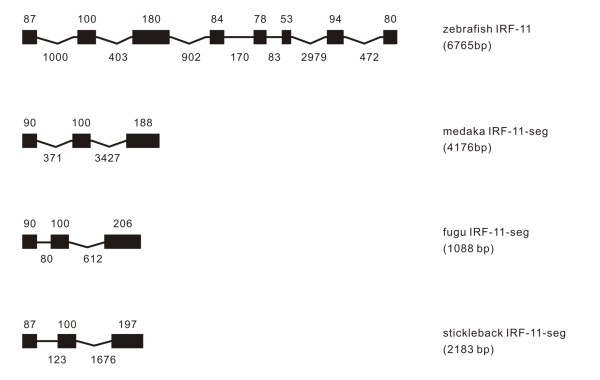
**Genomic structure of IRF-11 in teleost fish**. IRF-11 was only found in teleost, with the full length sequence available for zebrafish IRF-11. The gene name, exon-intron organization, and the fish species are expressed in a same way as in Figures 1 and 2.

Genomic organizations of IRF- 1 to 11 in vertebrates were illustrated in Figures 1, 2, 3, 4, 5, 6, 7, 8, 9, 10, 11. IRF-1 was identified in all vertebrates examined in the present study. The size of genomic structure of IRF-1 varied from 2163 to 9028 bp, with the longest observed in anole lizard, and shortest in stickleback (Figure 1). The IRF-1 all had a nine exon and eight intron structure in genome, except that in zebrafish eight exons were present. It is interesting to note that the size of the first two exons is the same in all vertebrates, and the first three is the same from frog to mammal. The first four exons were also the same in size in chicken and mammals, with the equal size in the seventh and eighth exons in mammals (Figure 1).

The intronic architecture of the examined IRFs is listed in Table 1. All intron phases are well conserved in IRF-1 with introns 1, 4, 7 in phase 0, introns 2, 3, 5, 6, and 8 in phase 1, except that in zebrafish, the third intron in other vertebrates might have lost in zebrafish IRF-1, as also indicated in the exon-intron organization of zebrafish IRF-1 (Figure 1).

**Table 1:** Distribution in intron phases of IRF coding region in vertebrates and non-vertebrate deuterostomes, including human, mouse, dog, chicken, anole lizard, frog, zebrafish, stickleback, sea squirt, lancet, sea uchin and acorn worm


**Gene**	**Start codon**	**Intron number**												
		
		**1**	**2**	**3**	**4**	**5**	**6**	**7**	**8**	**9**	**10**	**11**	**12**	**13**

human IRF-1	*	0	1	1	0	1	1	0	1	**◆**				

mouse IRF-1	*	0	1	1	0	1	1	0	1	**◆**				

dog IRF-1	*	0	1	1	0	1	1	0	1	**◆**				

chicken IRF-1	*	0	1	1	0	1	1	0	1	**◆**				

anole lizard IRF-1	*	0	1	1	0	1	1	0	1	**◆**				

frog IRF-1	*	0	1	1	0	1	1	0	1	**◆**				

zebrafish IRF-1	*	0	1	0	1	1	0	1	**◆**					

stickleback IRF-1	*	0	1	1	0	1	1	0	1	**◆**				

human IRF-2	*	0	1	1	0	1	1	0	**◆**					

mouse IRF-2	*	0	1	1	0	1	1	0	**◆**					

dog IRF-2	*	0	1	1	0	1	1	0	**◆**					

chicken IRF-2	*	0	1	1	0	1	1	0	**◆**					

anole lizard IRF-2	*	0	1	0	1	0	1	1	0	**◆**				

frog IRF-2	*	0	1	1	0	1	1	0	**◆**					

zebrafish IRF-2-seg	*	0	1	1	0	1	1	0						

stickleback IRF-2-seg	*	0	1	1	0	1	1	1	0					

human IRF-3	*	0	1	0	1	1	0	**◆**						

mouse IRF-3	*	0	1	0	1	1	0	**◆**						

dog IRF-3-seg			1	0	1	1	0	**◆**						

anole lizard IRF-3	*	0	1	0	1	0	1	1	0	0	**◆**			

frog IRF-3	*	0	1	0	1	1	1	1	0	**◆**				

zebrafish IRF-3	*	0	1	1	2	2	0	0	**◆**					

stickleback IRF-3	*	0	1	0	1	1	1	2	1	0	**◆**			

human IRF-4	*	0	1	0	1	1	1	0	**◆**					

mouse IRF-4	*	0	1	0	1	1	1	0	**◆**					

dog IRF-4-seg		0	0	1	0	1	1	1	0	**◆**				

chicken IRF-4	*	0	1	0	1	1	1	0	**◆**					

anole lizard IRF-4-seg		0	1	0	1	1	1	0	**◆**					

frog IRF-4-seg	*	0	0	0	2	1	0	0						

zebrafish IRF-4-c2	*	0	1	0	1	1	1	0	**◆**					

zebrafish IRF-4-c20	*	0	0	1	0	1	1	1	0	**◆**				

zebrafish IRF-4-scafNA1075-seg				1	2	0	0	0	1	**◆**				

stickleback IRF-4-1	*	0	2	0	1	0	1	1	1	0	**◆**			

stickleback IRF-4-2	*	0	1	0	1	1	0	**◆**						

human IRF-5	*	0	1	0	1	1	1	0	**◆**					

mouse IRF-5	*	0	1	0	1	1	1	0	**◆**					

dog IRF-5-seg	*	0	1	0	1	1	1	0						

chicken IRF-5-seg					1	1	1	0						

anole lizard IRF-5-seg					1	0								

frog IRF-5	*	0	1	0	1	1	1	0	**◆**					

zebrafish IRF-5	*	0	0	1	0	1	1	1	0	**◆**				

stickleback IRF-5	*	0	1	0	2	1	1	1	0	**◆**				

human IRF-6	*	0	1	1	1	1	0	**◆**						

mouse IRF-6	*	0	1	1	1	1	0	**◆**						

dog IRF-6	*	0	1	1	1	1	0	**◆**						

chicken IRF-6	*	0	1	1	1	1	1	0	**◆**					

anole lizard IRF-6	*	0	1	1	1	1	0	0	**◆**					

frog IRF-6-1	*	0	1	1	1	1	0	**◆**						

frog IRF-6-2	*	0	1	1	1	1	0	**◆**						

zebrafish IRF-6	*	0	1	1	1	1	0	**◆**						

stickleback IRF-6-seg	*	0	1	1	1	1	0	0						

human IRF-7	*	2	0	1	0	1	1	1	1	0	**◆**			

mouse IRF-7	*	2	0	1	0	1	1	1	0	**◆**				

dog IRF-7-seg							1	0	0	**◆**				

chicken IRF-7	*	2	0	1	2	0	1	1	0	0	0	1	0	**◆**

anole lizard IRF-7	*	2	0	1	0	1	1	1	0	**◆**				

frog IRF-7-seg	*	0	0	0	0	1	0	0						

zebrafish IRF-7	*	2	0	1	0	1	1	1	0	**◆**				

stickleback IRF7-seg			0	1	0	0	1	0	1	0	**◆**			

human IRF-8	*	0	1	0	1	1	1	0	**◆**					

mouse IRF-8	*	0	1	0	1	1	1	0	**◆**					

dog IRF-8	*	0	1	0	1	1	1	0	**◆**					

chicken IRF-8	*	0	1	0	1	1	1	0	**◆**					

anole lizard IRF-8	*	0	1	0	1	1	0	**◆**						

frog IRF-8-seg			1	0	1	1	1	0	**◆**					

zebrafish IRF-8	*	0	1	0	1	1	1	0	**◆**					

stickleback IRF-8	*	0	1	0	1	1	1	0	**◆**					

human IRF-9	*	0	1	0	1	1	1	0	**◆**					

mouse IRF-9	*	0	1	0	1	1	1	0	**◆**					

dog IRF-9	*	0	1	0	1	1	1	0	0	**◆**				

anole lizard IRF-9	*	0	1	0	1	1	1	0	**◆**					

frog IRF-9-seg			1	0	1	1	1	1	0	**◆**				

zebrafish IRF-9	*	0	0	1	0	1	1	1	2	**◆**				

stickleback IRF-9-seg	*	0	0	0	1	0	0	0	1	0	0			

dog IRF-10-seg			0	0	0	1	1	1	1	1	1	0	0	

chicken IRF-10-seg			1	0	1	1	1	0	**◆**					

anole lizard IRF-10-seg			1	0	2	1	2	1	0	0				

frog IRF-10-seg			0	1	1	1	1	0						

zebrafish IRF-10	*	0	1	0	1	1	1	0	**◆**					

stickleback IRF-10-seg	*	0	1	0	1	0	1	0	0					

zebrafish IRF-11	*	0	1	1	1	1	0	1	**◆**					

medaka IRF-11-seg	*	0	1	0										

fugu IRF-11-seg	*	0	1	0										

stickleback IRF-11-seg			0	1	0									

sea squirt IRF-like-scaf_162	*	2	0	1	0	0	0	0	0	1	**◆**			

sea squirt IRF-like-3q-1	*	0	1	1	0	1	**◆**							

sea squirt IRF-like-3q-2	*	0	1	1	1	1	**◆**							

sea squirt IRF-like-3q-3	*	0	1	1	1	1	**◆**							

sea squirt IRF-like-12q	*	0	1	1	1	0	1	0	0	0	0	**◆**		

sea squirt IRF-like-14p	*	0	1	1	0	1	1	**◆**						

sea squirt IRF-like-seg-1	*	0	1	1	0	0	1							

sea squirt IRF-like-seg-2			1											

sea squirt IRF-like-seg-3	*	1	1											

lancet IRF-like-scaf_187-1-seg	*	0	1	1	0	1	2	2	1	0	1			

lancet IRF-like-scaf_187-2	*	2	2	1	0	1	0	**◆**						

lancet IRF-like-scaf_187-3	*	0	2	1	**◆**									

lancet IRF-like-scaf_7	*	0	0	1	1	1	1	0	**◆**					

lancet IRF-like-scaf_12	*	0	1	**◆**										

lancet IRF-like-scaf_27	*	0	1	2	1	2	2	0	1	**◆**				

lancet IRF-like-scaf_48	*	0	1	**◆**										

lancet IRF-like-scaf_136	*	0	2	0	2	2	**◆**							

lancet IRF-like-scaf_172	*	0	1	1	1	**◆**								

lancet IRF-like-scaf_196	*	0	1	1	1	**◆**								

sea urchin IRF-like-1	*	2	1	1	1	0	1	1	**◆**					

sea urchin IRF-like-2	*	2	0	1	0	1	0	1	1	1	2	**◆**		

acorn worm IRF-like-1	*	2	1	2	1	0	1	1	0	1	1	**◆**		

acorn worm IRF-like-2	*	2	0	0	1	0	1	1	1	2	**◆**			

acorn worm IRF-like-3-seg		1	2											

* indicating start coden; The numbers 0, 1, 2 indicating the intron phase, and phase 0 indicating that intron lies between two codons, phase 1 between the first and second positions of a codon, phase 2 between the second and third positions of a codon; **◆ **indicating stop coden.

#### IRF-2

The genomic structure of IRF-2 has a range of 5205 to 52724 bp in length from fish to mammals, with the shortest observed in stickleback (Figure 2). The first three exons are quite conserved in size in all the vertebrates, except that the third exon in other vertebrates might have split into two exons in anole lizard. The other remaining exons were either identical in size, e.g., the two 47 bp exons and the 165 bp exons, or varied in a small range, e.g., from 112 to 118 bp in the fifth exon for all vertebrates except in fish, but in the sixth exon in anole lizard (Figure 2). However, the structure of IRF-2 in stickleback differed from other vertebrates, with the presence of nine exons. Analyses of exon sizes indicated that the sixth exon in other vertebrates might have split into two exons in stickleback (Figure 2).

Analyses of intron number and phase revealed a high degree of conservation in IRF-2 genes in vertebrates. However, the third and the sixth exons in other vertebrates were interrupted by a new phase 0 and phase 1 intron in anole lizard and stickleback, respectively (Table 1). Comparisons of the exon-intron structure and the intron phase between IRF-1 and IRF-2 in vertebrates imply that they might have been derived from a common ancestor.

#### IRF-3

The IRF-3 genomes varied considerably in their size, ranging from 2868 to 14802 bp and in the exon-intron organization (Figure 3). A conserved structure was observed in the size and organization for IRF-3 in human and mouse. However, it appears possible that the first exon is missing in dog IRF-3 when compared with that in human and mouse. With further searching, sequence gaps were found in the upstream of coding region in dog IRF-3; so whether the first exon is missing in dog IRF-3 requires further sequencing work. In zebrafish and frog, the IRF-3 had eight and nine exons respectively, and in both stickleback and anole lizard it has ten exons (Figure 3). Surprisingly, IRF-3 was not found in chicken.

Despite the variation in the exon-intron organization, phases for the first four introns of IRF-3 were the same in vertebrates from fish to mammals, except the difference in phases of the third and fourth introns in zebrafish IRF-3 and the sequence gap in dog IRF-3 (Table 1).

#### IRF-4

The genome of IRF-4 had a high degree of similarity in vertebrates from anole lizard to mammals in terms of the number of exons and their size (Figure 4). Several exons, such as the second, the fifth and sixth exons were of the same size in anole lizard, chicken, mouse and human. The size of exons was completely the same in human and mouse except the last one, and the IRF-4 in anole lizard and chicken was quite similar in the number of exons and their size. IRF-4 in dog had an additional exon, and the first two exons in dog might have been resulted from the separation of the first exon in other mammals. The genome of IRF-4 in frog and fish showed marked diversification when compared with IRF-4 in other vertebrates, with the presence of seven exons in frog.

Unexpectedly, three IRF-4-like genes were identified in zebrafish, which were named as the followings: fish species abbreviation-IRF-4-chromosome location-percent identity to human IRF-4-public protein id. For example, ddIRF-4-chr2-60.7%-EN6560, indicates zebrafish IRF-4 located on chromosome 2 with 60.7% identity to human IRF-4 and Ensembl database id ENSDARG0000006560, and other two IRF-4 were named as: ddIRF-4-chr20-1-52.0%-EN55374, ddIRF-4-scafNA1075-36.3%-EN35766. However, only two IRF-4 genes were observed in other teleost fish, such as in stickleback, gaIRF-4-groupIII-56.8%-EN16461, gaIRF-4-groupVIII-52.7%-EN4966; and in medaka, olIRF-4-chr4-56.7%-EN12712, olIRF-4-chr17-44.7%-EN17242; and in fugu, trIRF-4-scaf107-49.7%-EN2946, and trIRF-4-scaf305-52.5%-EN11568. The three IRF-4 genes in zebrafish varied in their size and genome organization, and were remarkably different from their mammalian counterparts in terms of exon sizes and intron phases (Table 1) although the whole gene structure of ddIRF-4-scafNA1075 could not be identified due to the sequence gaps in the upstream of coding region. However, the stickleback IRF-4-2 was probably comparable with the zebrafish IRF-4-C2 (Figure 2).

The intron phases were quite conserved in IRF-4 genes from anole lizard to mammals with introns 1, 3 and 7 in phase 0, and introns 2, 4, 5, 6 in phase 1 except the bias in dog which might have resulted from the insertion of the first exon in other vertebrates by a phase 0 intron. Of particular interest is the variation in intron phases in multi-copy genes of IRF-4 in teleost fish (Table 1).

#### IRF-5

One IRF-5 gene was found in vertebrates from fish to mammals (Figure 5). IRF-5 in mammals was comparable in genome structure and exon size. The frog IRF-5 had also eight exons as their mammalian counterparts, but the zebrafish IRF-5 had nine exons, with the size of the first two exons equivalent to the size of the first exon in frog IRF-5. The stickleback IRF-5 also had nine exons, thus comparable with the zebrafish. The IRF-5 in anole lizard was located in scaffold_7080, but was too short to characterize its gene structure. In chicken, IRF-5 was located at an unmapped region, with incomplete sequence information (Figure 5).

Comparisons of vertebrate IRF-5 exon/intron boundaries revealed a common intron position distribution from frog to mammals, with the introns 1, 3 and 7 in phase 0, and introns 2, 4, 5, 6 in phase 1 (Table 1), despite that most exons could not be characterized in the IRF-5 in anole lizard and chicken because of the incomplete genome sequences. The analyses of the genome and intron phase revealed that the first exon in other vertebrates was interrupted by a phase 0 intron in IRF-5 of zebrafish; and in addition, the IRF-5 in stickleback shared some characters in intron phases with IRF-5 in other vertebrates (Table 1).

#### IRF-6

The genome organization and exon sizes were quite conserved for IRF-6 in vertebrates from fish to mammals (Figure 6). In general, the gene consisted of 7 exons with 6 intervening introns, except that in chicken the IRF-6 had an eight exon structure, with the second exon in other vertebrate split into two exons (Figure 6). It is worth emphasizing that frog has two identical IRF-6 genes (Figure 6).

The intron positions of IRF-6 were strictly conserved in vertebrates from fish to mammals. The first and the last introns are in phase 0, while all other introns are in phase 1 (Table 1). It is interesting to note that the two IRF-6 genes in frog have the same size and the same position for each homologous exons and introns respectively, with the difference only in the size of introns (Table 1 and Figure 6).

#### IRF-7

The genome size of IRF-7 varied considerably from 2412 bp in mouse to 24469 bp in anole lizard. The size of exons and also of introns was variable. However, the IRF-7 had ten exons in human, nine in mouse, anole lizard, while it had thirteen exons in chicken. However, the chicken IRF-7 had sequence gaps in database, and IRF-7 was incomplete in dog and in frog genome databases (Figure 7).

Surprisingly, the IRF-7 differed in the phase position from other IRF members, in having a phase 2 intron, not a phase 0 intron as in other IRF members, separating the first two exons which encode the DBD region, the most conserved region in IRFs. The number of introns and intron phases varied considerably in different IRF-7 genes.

#### IRF-8

The IRF-8, in general, had a similar exon-intron organization in vertebrates from fish to mammal, with eight exons and seven introns (Figure 8), despite that in anole lizard and frog the IRF-8 had seven exons. The comparison of these exons may indicate that the merged-together of two exons might have occurred in anole lizard. The first exon could not be identified due to the sequence gaps in the upstream of frog IRF-8 coding region (Figure 8).

Among eight IRF-8 sequences from fish to mammal, the intron phases had exactly the same distribution. The classes of introns 1, 3, and 7 are in phase 0, leaving the others in phase 1 (Table 1), except that the fifth intron in other IRF-8 might have lost in IRF-8 in anole lizard (Table 1 and Figure 8).

#### IRF-9

IRF-9 was present in fish, frog, anole lizard, and mammals, but not in chicken (Figure 9). It was comparable in the whole length, and exon number and size in mammals, although the dog IRF-9 had two closely adjacent exons at last separated by only 4 bp (GCTT), which is not consistent with the GT/AG sequence at the donor and acceptor sites of RNA splicing (Figure 9). The anole lizard IRF-9 consisted of eight exons with seven introns as its counterparts in mammals but with larger variation in the size of introns. In zebrafish and stickleback, the IRF-9 had nine and ten exons, respectively; but they were quite comparable. Surprisingly, the Basic Local Alignment Search Tool (BLAST) results of predicted frog IRF-9 protein had a lack of first sixty amino acids in the IRF DBD region, which may require further study (Figure 9).

The distribution in intron phases for IRF-9 was quite comparable between mammals and anole lizard, with the introns 1 and 3 and the last one in phase 0 and introns 2, 4, 5, 6 in phase 1. However, the similar distribution in intron phases and the comparable exon sizes between IRF-8 and IRF-9 may imply, at least partially, that these two IRFs have a common origin. On the other hand, IRF-9 in lower vertebrates showed a high level of variation in intron phases (Table 1).

#### IRF-10

IRF-10 was not found in human and mouse, but at least an eleven-exon genome was present in dog (Figure 10). The zebrafish IRF-10 had eight exons and seven introns. However, the IRF-10 in other vertebrates was all incomplete. Nevertheless, the identified intron-exon structure in frog showed considerable variation when compared with other orthologues in other vertebrates (Figure 10). It is also quite impossible to summarize any pattern in the distribution of the intron phases (Table 1).

#### IRF-11

A new IRF family differing from previously-known ten members was identified only in teleost fish (Figure 11). The zebrafish IRF-11 had an eight exon structure in genome, while the IRF-11 in other teleost fish had an incomplete sequence data. Above all, these IRF-11 had the first exons similar to those in IRF-1 and IRF-2. Due to the incomplete sequences, the intron phase distribution was only analyzed for zebrafish IRF-11, with the findings of introns 1 and 6 in phase 0 and all others in phase 1 (Table 1).

### IRF-like genes in other deuterostomes

**Figure 12:**
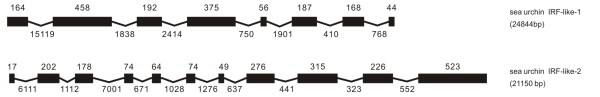
**Predicted genomic structure of IRF-like genes in the sea urchin *Strongylocentrotus purpuratus***.

**Figure 13:**
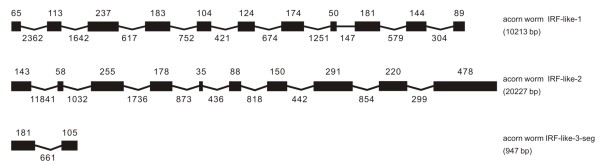
**Predicted genomic structure of IRF-like genes and IRF-containing sequence segment in the acorn worm *Saccoglossus kowalevskii***. The sequence segment was indicated with -seg in the gene name, as also listed in Table 1 and in Additional file 2.

**Figure 14:**
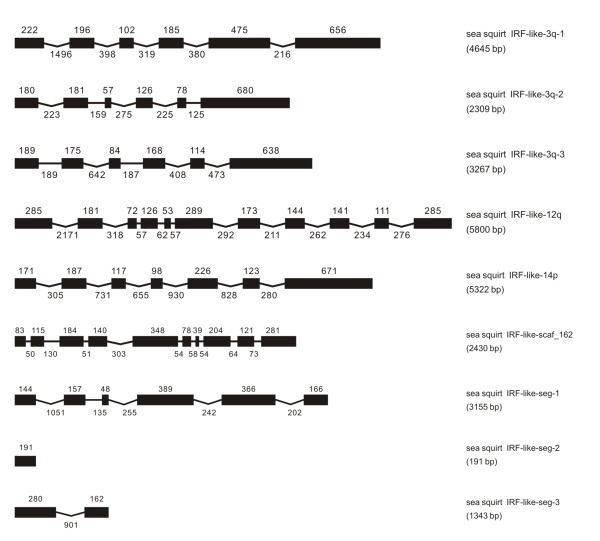
**Predicted genomic structure of IRF-like genes and IRF-containing sequence segments in the sea squirt *Ciona intestinalis***. The sequence segment was indicated with -seg in gene names as also in Table 1 and in Additional file 2.

**Figure 15:**
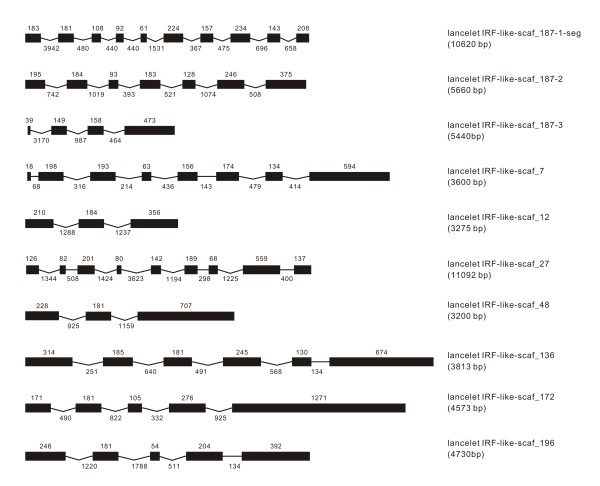
**Predicted genomic structure of IRF-like genes and IRF-containing sequence segment in the lancelet *Branchiostoma floridae***. The segment was indicated with -seg, and also in Table 1 and in Additional file 2.

The survey of several non-vertebrate deuterostome genomes may have broad implication in understanding the primitive state of vertebrate nervous, immune, and even cardiovascular systems [20,21]. Recent studies have revealed several types of immune-related gene families in these deuterostomes, such as Toll-like receptors and Ig superfamily, providing insight into the origin of immune system in chordates and vertebrates [22-24]. Despite a recent effort to identify IRF members in invertebrates [25], any possible IRF genes were identified in available databases of deuterostomes in the present study. Two IRF-like genes were identified in sea urchin *Strongylocentrotus purpuratus *(Figure 12), three in acorn worm *Saccoglossus kowalevskii *(Figure 13), nine in sea squirt *Ciona intestinalis *(Figure 14) and ten in lancelet *Branchiostoma florida *(Figure 15). Some of these genes were just sequence segments containing IRF DBD domains (Figures 13, 14, 15). The genomic structure of these IRF-like genes was further predicted. The genome organization varied among these genes, with little similarity detected. They showed almost no similarity to any IRF members of vertebrates in genome organization (Figures 1, 2, 3, 4, 5, 6, 7, 8, 9, 10, 11, 12, 13, 14, 15) and also in sequence (Additional file 1). These IRF-like genes also had almost no similarity in intron phases, with the only exception in sea squirt that the IRF-like-3q-2 and IRF-like-3q-3 have the same intron phases and comparable gene structure, differing from sea squirt IRF-like-3q-1 (Table 1).

### Synteny analyses

**Figure 16:**
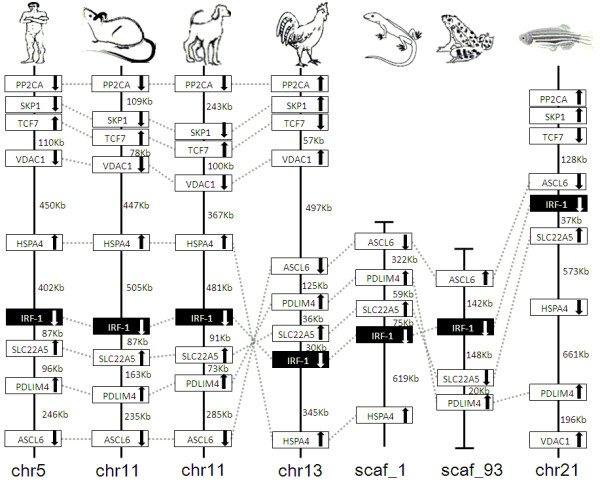
**IRF-1 gene loci in human, mouse, dog, chicken, anole lizard, frog and zebrafish**. Conserved genes are boxed, and vertical lines represent chromosomes; arrows indicate the transcription orientation of genes. Numbers on the side of the lines indicate the distance between genes. PP2CA: protein phosphatase 2 (formerly as 2A), catalytic subunit; SKP1: S-phase kinase-associated protein 1A; TCF7: transcription factor 7 (T-cell specific, HMG-box); VDAC1: voltage-dependent anion channel 1; HSPA4: heat shock 70 kDa protein 4; SLC22A5: solute carrier family 22 (organic cation transporter), member 5; PDLIM4: PDZ and LIM domain 4; ASCL6: acyl-CoA synthetase long-chain family member 6.

**Figure 17:**
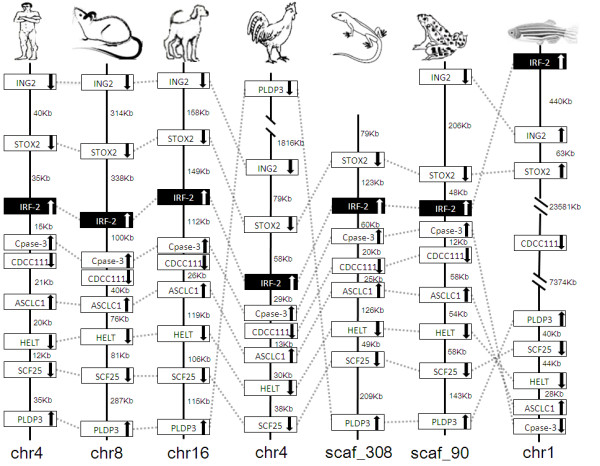
**IRF-2 gene loci in human, mouse, dog, chicken, anole lizard, frog and zebrafish**. The boxed genes, vertical lines and numbers on the side of lines in this figure and in following Figures 18 to 24 are as same as indicated in Figure 16. ING2: Inhibitor of growth protein 2; STOX2: storkhead box 2; Cpase-3: Caspase-3; CDCC111: Coiled-coil domain-containing protein 111; ASCLC1: acyl-CoA synthetase long-chain family member 1; HELT: Hey-like transcription factor; SCF25: ADP/ATP translocase 1/Solute carrier family 25 member 4; PLDP3: PDZ and LIM domain protein 3.

**Figure 18:**
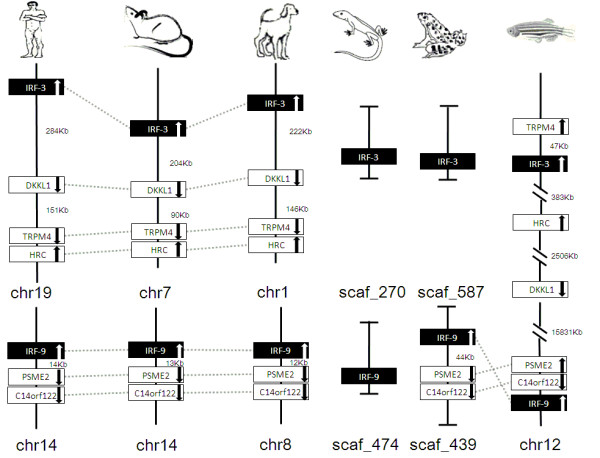
**IRF-3 and IRF-9 gene loci in human, mouse, dog, anole lizard, frog and zebrafish**. DKKL1: dickkopf-like 1; TRPM4: transient receptor potential cation channel, subfamily M, member 4; HRC: histidine rich calcium binding protein; PSME2: proteasome (prosome, macropain) activator subunit 2 (PA28 beta); C14orf122: chromosome 14 open reading frame 122.

**Figure 19:**
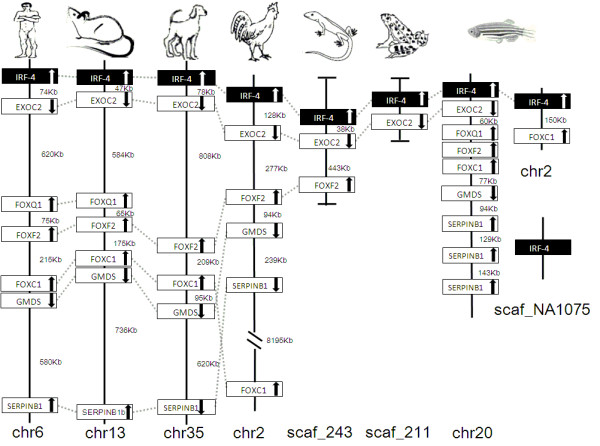
**IRF-4 gene loci in human, mouse, dog, chicken, anole lizard, frog and zebrafish**. EXOC2: exocyst complex component 2; FOXQ1: forkhead box Q1; FOXF2: forkhead box F2; FOXC1: forkhead box C1; GMDS: GDP-mannose 4,6-dehydratase; SERPINB1: serpin peptidase inhibitor, clade B (ovalbumin), member 1.

**Figure 20:**
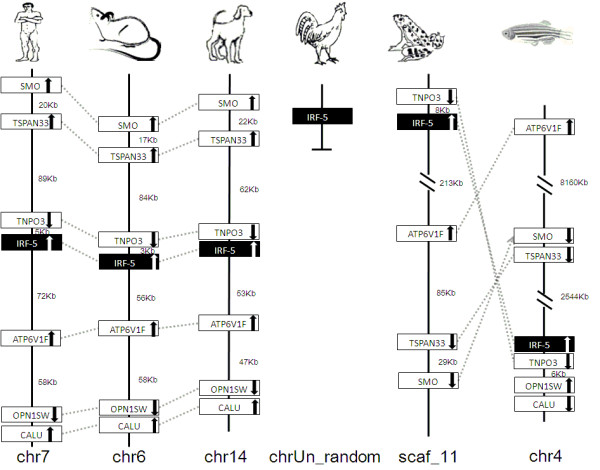
**IRF-5 gene loci in human, mouse, dog, chicken, anole lizard, frog, and zebrafish**. SMO: smoothened homologues; TSPAN33: tetraspanin 33; TNPO3: transportin 3; ATP6V1F: ATPase, H+ transporting, lysosomal 14 kDa, V1 subunit F; OPN1SW: opsin 1 (cone pigments), short-wave-sensitive (color blindness, tritan); CALU: calumenin.

**Figure 21:**
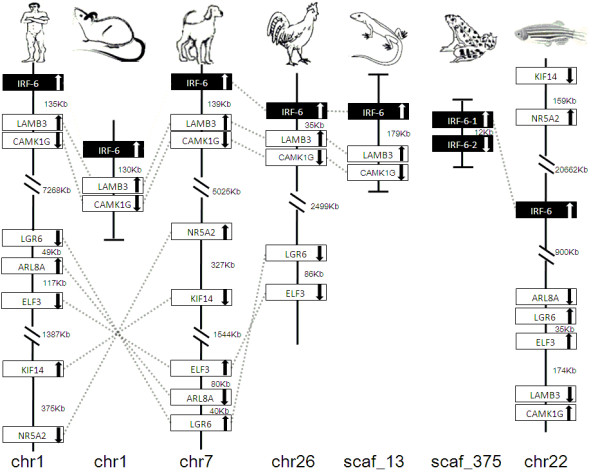
**IRF-6 gene loci in human, mouse, dog, chicken, anole lizard, frog and zebrafish**. LAMB3: laminin, beta 3; CAMK1G: calcium/calmodulin-dependent protein kinase IG; LGR6: leucine-rich repeat-containing G protein-coupled receptor 6; ARL8A: ADP-ribosylation factor-like 8A; ELF3: E74-like factor 3; KIF14: kinesin family member 14; NR5A2: nuclear receptor subfamily 5, group A, member 2.

**Figure 22:**
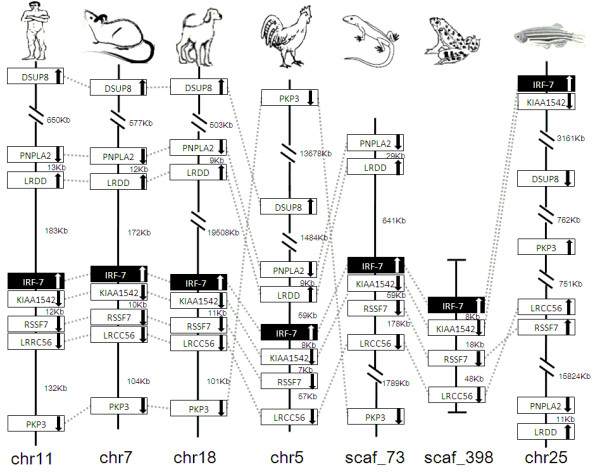
**IRF-7 gene loci in human, mouse, dog, chicken, anole lizard, frog and zebrafish**. DSUP8: dual specificity phosphatase 8; PNPLA2: patatin-like phospholipase domain containing 2; LRDD: leucine-rich repeats and death domain containing; KIAA1542: CTD-binding SR-like protein rA9, RSSF7: Ras association (RalGDS/AF-6) domain family 7, LRRC56: leucine rich repeat containing 56, PKP3: plakophilin 3

**Figure 23:**
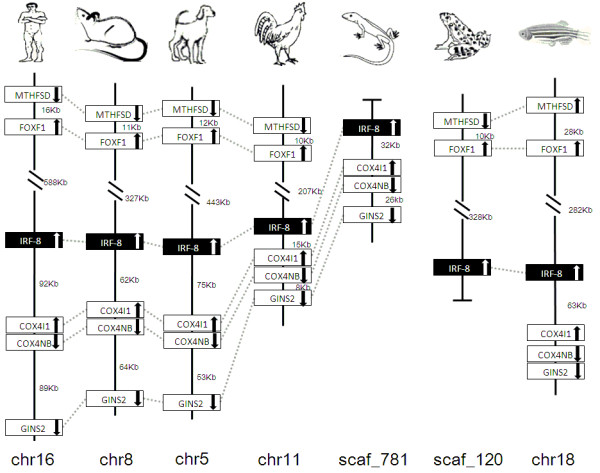
**IRF-8 gene loci in human, mouse, dog, chicken, anole lizard, frog and zebrafish**. MTHFSD: methenyltetrahydrofolate synthetase domain containing; FOXF1: forkhead box F1; COX4I1: cytochrome c oxidase subunit IV isoform 1, COX4NB: COX4 neighbor; GINS2: GINS complex subunit 2 (Psf2 homologue).

**Figure 24:**
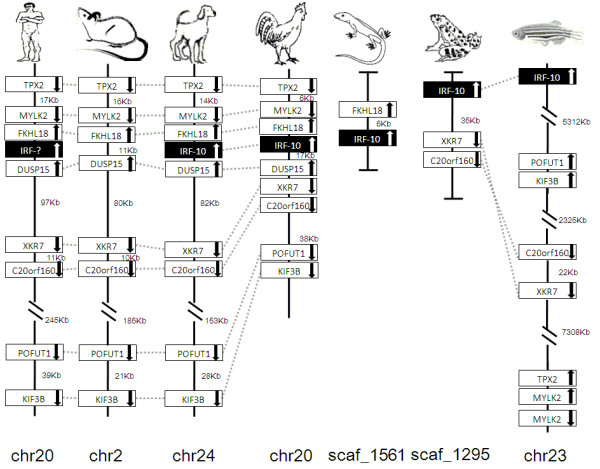
**IRF-10 gene loci in human, mouse, dog, chicken, anole lizard, frog and zebrafish**. TPX2: microtubule-associated protein homologue (*Xenopus laevis*); MYLK2: myosin, light polypeptide kinase 2, skeletal muscle; FLKHL18: forkhead-like 18 (*Drosophila*), DUSP15: dual specificity phosphatase-like 15; XKR7: X Kell blood group precursor related family member 7 homologue, C20orf160: chromosome 20 open reading frame 160, POFUT1: protein O-fucosyltransferase 1, KIF3B: kinesin family member 3B.

IRF members were located by using BLAST algorithm to chromosomes or to scaffolds in frog and anole lizard as their genome wide sequence data is rather limited (Figures 16, 17, 18, 19, 20, 21, 22, 23, 24). The transcription orientation was well conserved in all IRF members, i.e. from IRF-1 to IRF-10 in all clearly identified genomic sequences for the vertebrates (Figures 16, 17, 18, 19, 20, 21, 22, 23, 24). The two IRF-6 genes in frog, which were connected together, were transcribed in two adverse directions (Figure 21). It is further worth noticing that IRF-3 and IRF-9 were spread on a same chromosome in zebrafish (Figure 18).

There is certain degree of conservation in the IRF loci in vertebrates (Figures 16, 17, 18, 19, 20, 21, 22, 23, 24). The genes adjacent to IRF genes were comparable. The arrangement or the genes close to IRF genes were conserved to a large degree in human, mouse and dog, and chicken (Figures 16, 17, 18, 19, 20, 21, 22, 23, 24). Genome sequence data in anole lizard and frog was rather limited, and thus synteny analysis was less informative in these vertebrates. Genes in IRF gene loci in zebrafish were overall comparable to their mammalian counterparts: Some loci were very similar; for example, the first gene next to IRF-4, and IRF-4-C20 in fish and to IRF-7 was the same in all vertebrates, and the IRF-8 loci were quite similar in all vertebrates. But, the conserved genes may be arranged in a much longer distance on chromosomes in fish, e.g., in IRF-2, IRF-3, IRF-5 and IRF-10 loci, and/or in different positions above or below the IRF gene, e.g., in IRF-1 loci. Despite the absence of IRF-10 gene in human and mouse, a segment encoding 210 aa was identified in an arrangement conserved in others of dog and chicken (Figure 24).

The so-called macro-synteny, i.e., comparison of gene linkage on chromosomes between amphioxus, the lancelet *Branchiostoma floridae *and human has revealed the existence of 17 linkage groups in the lancelet [24], and the locations of IRF-like genes have been identified in individual genome linkage groups, in comparison with IRF genes in different chromosomes of human [25]. But, IRF-2 could not be traced in these possible linkage groups in the present study, although Nehyba et al. [25] hypothesized its existence in linkage groups 6.

### Percent identity and phylogeny

IRF- 1 to 9 in vertebrates were compared respectively with their counterparts in human by using Needleman-Wunsch global alignment. All identified IRFs in mouse and dog showed highest homology to their counterparts in human (Additional file 1). The percent identity appeared lower in lower vertebrates, and higher in higher vertebrates. Interestingly, some members such as IRF-2, IRF-4, IRF-6 and IRF-8, showed a higher degree of identity with their human counterparts (42.8% to 92.8%, 36.3% to 92.2%, 56.0% to 97.6, 31.8% to 91.8% respectively) (Additional file 1), implying that they may have some evolutionary conservation. Of all the IRF-10 identified in vertebrates, only avian IRF-10 showed some sequence similarity with the homologue in dog (41.3% identity, 49.9% similarity).

However, the percent identity of IRF-like genes or segments in non-vertebrate deuterostomes had less similarity to mammalian IRF members (Additional file 2). The highest similarity was below 43.6% between lancelet IRF-like-scaf187-2 and human IRF-8 (Additional file 2). In all of the four non-vertebrate deuterostomes, some identified or predicted IRF-like genes had a similarity above 30% to certain IRF members in human (Additional file 2).

**Figure 25:**
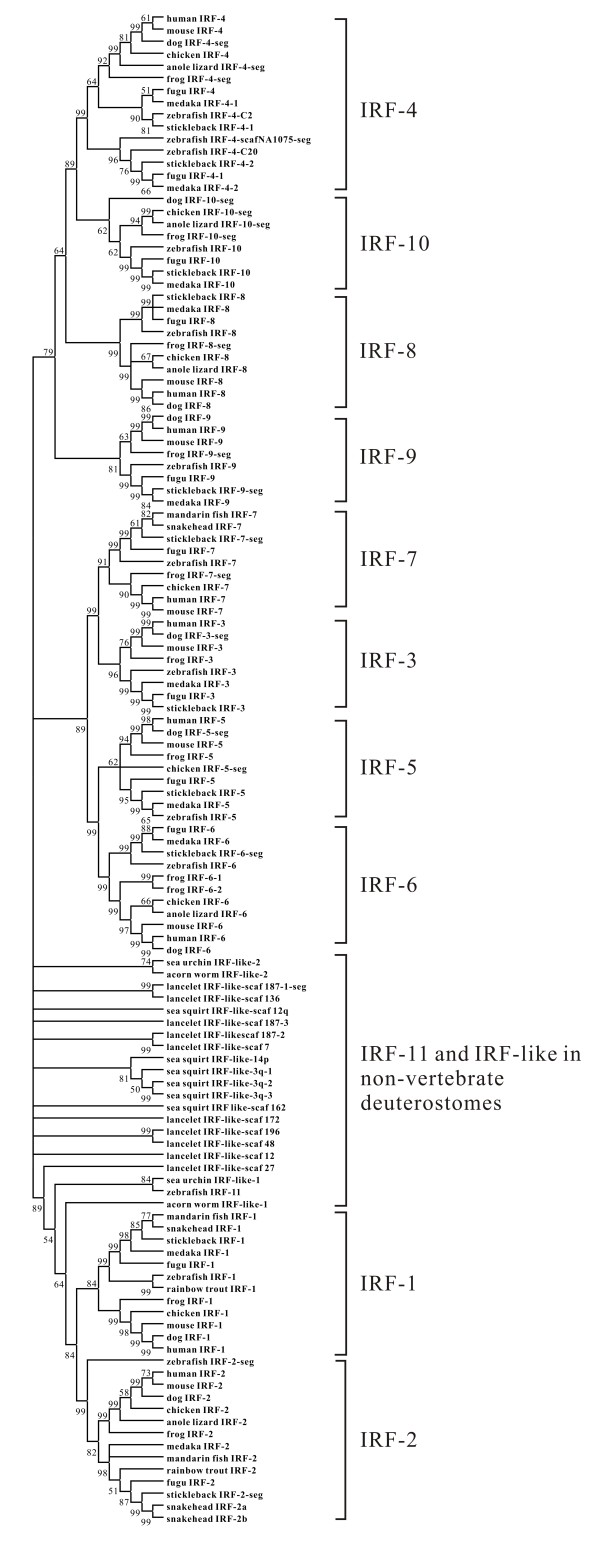
**Phylogenetic analysis of IRF members in chordates by using neighbor-joining method within Mega4.0 program**. Bootstrap values are indicated at nodes. Anole lizard IRF-5-seg, Dog IRF-7-seg and IRF-like gene segments in non-vertebrate deuterostomes were not included in the phylogenetic analysis due to the relatively short sequence lengths, and all IRF or -like genes shown in the tree are listed in Additional files 1 and 2.

The phylogenetic relationship of all IRF members including IRF-1 to IRF-11 in vertebrates and those IRF-like genes identified in all non-vertebrate deuterostomes in the preset study was shown in Figure 25. It is apparent that all well-characterized IRF members, i.e., those from IRF-1 throughout to IRF-10 were clustered into separate clades. None of IRF-like genes was clustered within the ten clades containing these already-known 10 IRF members (Figure 25). The IRF-like genes in lancelet and sea squirt were mostly clustered in each species. Interestingly, the acorn worm IRF-like-1, equivalent to SK1 in Nehyba et al. [25] (Additional file 2), was clustered with the clade containing IRF-1 and IRF-2. Furthermore, it is obvious that the zebrafish IRF-11 was clustered together with sea urchin IRF-like-1 (Figure 25).

## Discussion

All known IRF members, i.e., from IRF-1 to IRF-10 were identified in classes of vertebrates, i.e., piscine (except Chondrichthyans whose genomes are too scarce to analyze), amphibian, reptile, avian and mammal, despite that IRF-10 was not found in human and mouse, and that an additional IRF member was identified in teleost fish. The absence of IRF-3 and IRF-9 in chicken may be just a reflection of its incomplete genome sequences; alternatively these two genes may not exist in chicken at all. Overall, it is likely that vertebrates have a similar composition of IRF family members. In a recent study to investigate the evolution of IRF-like genes in invertebrates, Nehyba et al. [25] concluded that IRF family evolved distinctly in different taxonomical groups within Bilateria, as in several groups of animals the IRF family developed to a large number of family members. However, the comparable number of IRF members in classes of vertebrates may thus reflect a higher degree of conservation, and probably a similar pattern of evolution and function for these IRFs in vertebrates.

The complexity and diversity of immune systems in vertebrates increased throughout evolution, and new genes arose at various time points by gene fission and fusion [26], and retrotransposition and duplication during evolution [27]. The sequencing in whole genomes of various organisms has been providing opportunities to trace the origin of genes which comprise the immune system in vertebrates [28,29]. It is reported that the IRF genes originated with the appearance of mulitcellular animals are present in almost all metazoan groups. In sponges and placozoans, the number of IRF family members is two, and five in cnidarians; and in several groups of animals such as in molluscan, cephalochordate, tunicate and vertebrate, the number might have evolved independently [25].

IRF members in human may be separated into IRF-1 and IRF-4 subgroups, with the former having a C-terminal associated domain (IAD2) and the later a different C-terminal associated domain (IAD1) [3,30]. Using DBDs and IAD1 respectively, rather than the entire IRF proteins as in the present study, Nehyba et al. [25] were able to separate IRF-like genes into IRF-1 and IRF-4 groups in bilaterians, with four genes (as SK1, SP1, BF4, CI1) identified from each of the four species, acorn worm, lancelet (amphioxus), sea urchin and sea squirt being in the super group of IRF-1. In the present study, the acorn worm IRF-like-1, equivalent to SK1 (Additional file 2), has a close relationship with the big clade containing both IRF-1 and IRF-2, and the sea urchin IRF-like-1 (equivalent to SP1) is clustered with the clade containing IRF-1 and IRF-2. These two IRF-like genes may thus have a close evolutionary relationship with IRF-1 and IRF-2. The lancelet IRF-like-scaf_27 (equivalent to BF4) appeared relatively close with this clade. But, the CI1, equivalent to sea squirt IRF-like-seg-3 is just a segment (Figure 14) which was not included in the present phylogenetic study. However, all IRF members identified in vertebrates were clustered in four groups in the present phylogenetic tree, as reported by Nehyba et al. [10], i.e., IRF-1 group including IRF-1 and IRF-2, IRF-3 group including IRF3 and IRF7, IRF-4 group including IRF-4, IRF-8, IRF-9 and IRF-10, and IRF-5 including IRF-5 and IRF-6. It can be concluded at least tentatively that the phylogenetic relationship of IRF-like genes in non-vertebrate deuterostomes with those in vertebrates remains, to a large extent, unsolved. Further sequence analysis as well as possible function analysis may be required to understand the true phylogenetic relationship of these IRF family members in chordates. If the view that IRF family evolved distinctly in different taxonomical groups by Nehyba et al. [25] were true in different groups of chordates, it might be rather difficult to trace back the origin and the true phylogeny of IRF members, unless enormous sequence data from a variety of organisms are gathered.

However, using the 17 chordate linkage groups constructed by Putnam et al. [24] for the comparison of lancelet and vertebrate genes, Nehyba et al. [25] located 10 human IRF genes, including a segment as syntenic to other vertebrate IRF-10 in the present study, to four linkage groups, each of which was related to one of the four groups of IRF family in vertebrates. But, they also considered that the distinct IRF genes in lancelet and their location in eight different linkage groups may simply represent a pattern of extensive independent evolution, and at the meantime they recognized that the lancelet IRF-like-scaf_27 gene (as BF4 in their study), which has some relationship with the IRF-1 and IRF-2 clade in the present study and was located in the sixth linkage group to which the human IRF-1 was traced, may be considered as the most likely gene linked to the predecessor of vertebrate IRF-1 gene.

**Figure 26:**
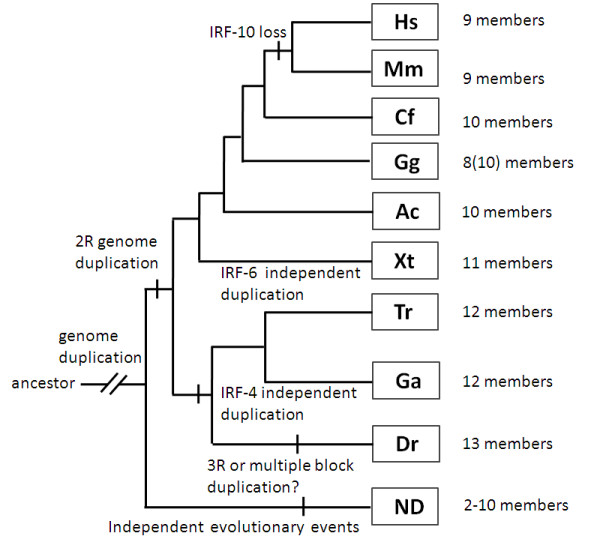
**A cladistic model for the evolution of IRF family members**. Two rounds of duplication of the whole genome are inferred to have occurred by the base of vertebrates, and tandem duplication, perhaps even an extra round of tetraploidization in zebrafish lineage. Subsequent independent gene duplication and/or loss events were marked on the tree. Hs: human *Homo sapiens*; Mm: mouse *Mus musculus*; Cf: dog *Canis familiaris*; Gg: chicken *Gallus gallus*; Ac: anole lizard *Anolis carolinensis*; Xt: frog *Xenopus tropicalis*; Tr: fugu *Takifugu rubripes *Ga: stickleback *Gasterosteus aculeatus*; Dr: zebrafish *Dario rerio*; ND: non-vertebrate deterostomes.

It is now generally agreed that a two-fold whole genome duplication might have occurred in early vertebrates after their separation from other deuterostomes [31]. Nehyba et al. [25] suggested that the generation of ten IRF genes in vertebrates might be resulted also from the duplication. On the basis of our data, a plausible model was proposed for the evolutionary history of IRF gene family (Figure 26). In this model, all IRF genes were assumed to have evolved from a common ancestor that might have undergone duplication and subsequent mutation or rearrangement prior to the divergence of urochordates, resulting in multiple IRF genes. Subsequent gene duplication of these ancestral IRF genes in some chordates and vertebrates might represent two separate evolutionary events. In vertebrates, the IRF gene might have undergone additional duplication at the time before fish diverged from their vertebrate ancestor. Polyploidization in vertebrates may have promoted such innovation while vertebrate ancestors probably only possessed single copy of gene found now in multiple copies in vertebrates [32]. Moreover, eleven to thirteen IRF genes were identified in jawed fish. Compared with other vertebrates, teleost fish have an additional member, IRF-11, which at least showed some similarity to the IRF-1 and IRF-2 in the first exons, indicating that genome, or gene and probably fragment duplication, as well as gene shuffling might have occurred in fish for the arising of this new member.

The eleventh member of IRF in fish has been reported in an early study [19], but all the IRF-11 identified previously is in fact the IRF-1 in the present study, as revealed by genome and synteny analyses in the present study. So, the IRF-11 identified in the present study is a real new member in the IRF family, and it has limited similarity with IRF-1 and IRF-2 as shown in their genome and in phylogenetic analysis.

Interestingly, there are two IRF-4 genes present in most teleost species and three in zebrafish, but only one in higher vertebrates, suggesting that in teleost fish, especially in zebrafish tandem gene duplication or additional independent chromosome duplication might have occurred [33]. The similarity in the genome organization and intron phases in IRF-1 and IRF-2 may indicate that these two genes might have evolved from a common ancestor, by at least partial duplication or gene shuffling; and this may also be case for IRF-8 and IRF-9. The genomic organization of some IRF genes, such as IRF-5 in zebrafish (the first two exons), the middle exons of IRF-6 in chicken and dog, and the IRF-8 in frog and anole lizard might have also been resulted from gene shuffling, as also revealed by intron phase analyses. Furthermore, the copy of another IRF-6 downstream of the first IRF-6 gene in scaffold_375 in frog also provides evidence for the independent gene duplication. Regarding the IRF-10, higher vertebrate lineages might have lost the gene due to redundancy of function or compensation by other genes during evolution, given the conserved synteny of the IRF-10 locus in the genomes of vertebrates.

Nevertheless, vertebrates in different classes exhibited a high degree of conservation in the number of IRF family members, implying that the IRF family members might have evolved from common ancestors with similar evolutionary mechanisms, although multiple genes of IRF-4 and IRF-6, and even an additional IRF family member have evolved in lower vertebrates and IRF-3 and IRF-9, and IRF-10 have lost in chicken, and human and mouse, respectively. The genomic organizations for IRF-1, IRF-2, IRF-6, IRF-8 and IRF-9 and IRF-4 in higher vertebrates may be quite conservative. On the other hand, the comparison of substantial local sharing of neighbouring genes in different species may enable the understanding of phylogenetic relationships among IRF members, or even infer the genome evolution. Comparisons of IRF-4, IRF-8, IRF-9 and IRF-10 orthologues pertaining regions in vertebrates showed that these four members were co-localized with the Fox family member. These instances, therefore, were consistent with the study on Fox family. It seems likely that these four members were initially generated by an ancestral duplication [34]. The presence of conserved synteny but the lack of gene linkage in IRF orthologous regions in teleost fish when compared with other vertebrates, may suggest that inversions were more prevalent than translocations in the evolution of IRF locus, and at least some regions in teleost fish genomes might have undergone substantial intra-chromosomal rearrangements over evolution. Evidence for this conclusion comes from the comparison of IRF-2, IRF-3, IRF-5, IRF-6, IRF-7 and IRF-10 pertaining chromosome regions in vertebrates. The orders of these loci within chromosome segments in zebrafish are substantially rearranged. However, when viewed in IRF syntenic map in human, mouse, dog and chicken, most genes have obvious counterparts in the syntenic region with the same order. In these instances, a higher level of conservation in synteny suggests that similar selective forces might have been operating since these lineages diverged.

## Conclusions

Ten members of IRF family, i.e., from IRF-1 to IRF-10 have been identified in vertebrates, although IRF-3 and IRF-9 were not found in chicken and IRF-10 not in human and mouse. However, lower vertebrates such as frog and fish have multiple genes of IRF-6 and IRF-4, respectively. Surprisingly, an additional number, IRF-11 was identified only in teleost fish. The genomes of some IRF family members, such as IRF-1, IRF-2, IRF-6, IRF-8 etc. have conserved structure in terms of exon-intron organization and also in the distribution of intron phases. The genes adjacent to IRF genes in vertebrates are quite comparable, especially in higher vertebrates; but it seems likely that conserved genes were spread in a much longer distance in chromosomes of zebrafish. The number of IRF family members in different classes of vertebrates might have been resulted from whole genome, or gene duplication, or even gene shuffling, and probably chromosome rearrangement especially in fish.

## Methods

### Database mining and genome analysis

Genomic sequences were downloaded from current assemblies within the Ensembl Database http://www.ensembl.org for human *Homo sapiens*, mouse *Mus musculus*, dog *Canis familiaris*, chicken *Gallus gallus*, anole lizard *Anolis carolinensis*, frog *Xenopus tropicalis*, zebrafish *Danio rerio*, stickleback *Gasterosteus aculeatus*, medaka *Oryzias latipes*, fugu *Takifugu rubripes*, and sea squirt *Ciona intestinalis*, the Department of Energy Joint Genome Institute database for lancelet (amphioxus) *Branchiostoma floridae *http://genome.jgi-psf.org/Brafl1/Brafl1.home.html, and National Center for Biotechnology Information http://www.ncbi.nlm.nih.gov for acorn worm *Saccoglossus kowalevskii*, sea urchin *Strongylocentrotus purpuratus *WGS sequences. Gene prediction was performed with FGENESH http://www.softberry.ru/berry.phtml and GENSCAN http://genes.mit.edu/GENSCAN.html. A quantitative sequence analysis with Hidden Markov Model (HMM) [35] was used to identify IRF genes. The DBD regions of IRF already dated in human and mouse were sorted according to their scores in HMMs trained on DBD motifs model and were used to search from the predicted gene database. All predicted IRF protein sequences were verified by BLASTP in the NCBI non-redundant protein sequence database and Ensembl database by using the BLASTP and TBLASTN programs, and any matches were used to refine prediction. The DNA region covering the query result of each predicted IRF protein coding region and the corresponding transcripts accessed by Ensemble database were extracted and the SIM4 program http://pbil.univ-lyon1.fr/sim4.php was used to reconstruct the exon-intron structure and calculate the intron phase. All IRFs and their genomic locations are listed in Additional files 1 and 2.

### Pairwise homology of all examined IRFs

Sequence comparisons of human IRF proteins and other putative IRFs mined from the examined species were performed with Needleman-Wunsch global alignment by using the needle program from the EMBOSS package with default parameters (Gap opening penalty 10.0, Gap extension penalty 0.5) [36], and the percent identity of the top-scoring pair was obtained (Additional files 1 and 2).

### Phylogenetic analysis of IRFs

For molecular phylogenetic analyses, protein sequences were first aligned using ClustalW [37]. Phylogenetic analyses were performed using the neighbor-joining method within the Mega4.0 program [38]. Data were analyzed using Poisson correction, and gaps were removed by pairwise deletion. The bootstrap values of the branches were obtained by testing the tree 10,000 times.

### Identification of conserved genes in IRF syntenic region from human, mouse, dog, chicken, anole lizard, frog and zebrafish and stickleback

To analyze the IRF syntenic region in vertebrates, human IRFs were used as anchor sites. Comparisons of the orthologues flanking the human anchor sites were accomplished by BLAST against the following NCBI genome assemblies: *Homo sapiens *(Build 36.3), *Mus musculus *(Build 37.1), *Canis familiaris *(Build 2.1), *Gallus gallus *(Build 2.1) on NCBI map viewer, and *Anolis carolinensis *(1.0), *Danio rerio *(Zv6) and *Xenopus tropicalis *(version 4.1) on Ensembl genome browser. Complete gene lists compiled from the syntenic region were obtained in these following regions: Human (chromosome5, 13137051-133589849; chromosome4, 184663214-186693650; chromosome9, 54346267-54860926; chromosome6, 336760-2787080; chromosome7, 128166672-128640622; chromosome1, 198263393-208046102; chromosome11, 384217-1549726; chromosome16, 84268781-85146317; chromosome14, 23678014-23705614; chromosome20, 29790565-30386475); Mouse (chromosome11, 51912326-54175041; chromosome8 46970839-48760512; chromosome7 52253030-52594343, chromosome13, 30841127-33186181; chromosome6, 29298119-29711359; chromosome1, 194979306-195196476; chromosome7, 148265677-149276175; chromosome8, 123105166-123632218; chromosome14, 56194511-56228867; chromosome2, 152673782-153157950); Dog (chromosome11, 23231763-25417744; chromosome16, 48072456-49811062; chromosome1, 109762805-110195996; chromosome35, 3765639-6182542; chromosome14, 10477159-10856486; chromosome7, 3729824-11424871; chromosome18, 28527750-48830961; chromosome5, 69307830-69996495; chromosome8, 7139778-7162946; chromosome24, 24192691-24676398); Chicken (chromosome13, 16330227-17520976; chromosome4, 40757647-41228574; chromosome2, 6766708-77050634; chromosome Un_random Contig1969; chromosome26, 316853-2993929; chromosome5, 1579936-17138835; chromosome11, 19359246-19654930; chromosome20, 9989995-10113464); Anole lizard (scaffold_1, scaffold_308, scaffold_270, scaffold_243, scaffold_7087, scaffold_13, scaffold_73, scaffold_781, scaffold_474, scaffold_1561); Frog (scaffold_93, scaffold_90, scaffold_587, scaffold_211, scaffold_11, scaffold_375, scaffold_398, scaffold_120, scaffold_439, scaffold_1295); Zebrafish (chromosome21, 44732965-48302889; chromosome1, 19605403-51285221; chromosome12, 3671869-4198128; chromosome2, 192032-182022; scaffold_NA1075, chromosome20, 19100202-31847658; chromosome4, 13813890-22022810; chromosome22, 198423-22346497; chromosome25, 5026720-25819085; chromosome18, 29472647-29955285; chromosome12, 22543362-22592525; chromosome23, 835503-15973618).

## Authors' contributions

HB and NP were primarily responsible for the designing, conduction of the study and wrote the manuscript. HB conducted the database searches and bioinformatics analysis. QZT contributed to the phylogenetic analysis. XZ conducted some searches. All author read and approved the final manuscript

## Supplementary Material

Additional file 1 **IRF genes identified in vertebrates**. Table I IRF genes identified in vertebrates from fish to mammals, including human, mouse, dog, chicken, anole lizard, frog, zebrafish, stickleback, fugu, medaka, mandarin fish, rainbow trout, snakehead. Click here for file

Additional file 2 **Homologues of IRFs in non-vertebrate deuterostomes**. Table II Sequence information of homologues of IRFs in non-vertebrate deuterostomes including sea squirt, lancelet, sea urchin and acorn worm. Click here for file

## References

[B1] Mamane Y Heylbroeck C Genin P Algarte M Servant MJ LePage C DeLuca C Kwon H Lin R Hiscott J Interferon regulatory factors: the next generation Gene 1999 237 1 14 10.1016/S0378-1119(99)00262-0 10524230

[B2] Colonna M TLR pathways and IFN-regulatory factors: to each its own Eur J Immunol 2007 37 306 309 10.1002/eji.200637009 17273997

[B3] Tamura T Yanai H Savitsky D Taniguchi T The IRF family transcription factors in immunity and oncogenesis Annu Rev Immunol 2008 26 535 584 10.1146/annurev.immunol.26.021607.090400 18303999

[B4] Taniguchi T Ogasawara K Takaoka A Tanaka N IRF family of transcription factors as regulators of host defense Annu Rev Immunol 2001 19 623 655 10.1146/annurev.immunol.19.1.623 11244049

[B5] Stellacci E Testa U Petrucci E Benedetti E Orsatti R Feccia T Stafsnes M Coccia EM Marziali G Battistini A Interferon regulatory factor-2 drives megakaryocytic differentiation Biochem J 2004 377 367 378 10.1042/BJ20031166 14505489 PMC1223861

[B6] Battistini A Interferon regulatory factors in hematopoietic cell differentiation and immune regulation J Interferon Cytokine Res 2009 29 765 780 10.1089/jir.2009.0030 19929577

[B7] Escalante CR Yie J Thanos D Aggarwal AK Structure of IRF-1 with bound DNA reveals determinants of interferon regulation Nature 1998 391 103 106 10.1038/34224 9422515

[B8] Fujita T Sakakibara J Sudo Y Miyamoto M Kimura Y Taniguchi T Evidence for a nuclear factor(s), IRF-1, mediating induction and silencing properties to human IFN-beta gene regulatory elements EMBO J 1988 7 3397 3405 2850164 10.1002/j.1460-2075.1988.tb03213.x PMC454838

[B9] Barnes B Lubyova B Pitha PM On the role of IRF in host defense J Interferon Cytokine Res 2002 22 59 71 10.1089/107999002753452665 11846976

[B10] Nehyba J Hrdlickova R Burnside J Bose HR Jr A novel interferon regulatory factor (IRF), IRF-10, has a unique role in immune defense and is induced by the v-Rel oncoprotein Mol Cell Biol 2002 22 3942 3957 10.1128/MCB.22.11.3942-3957.2002 11997525 PMC133824

[B11] Klein U Casola S Cattoretti G Shen Q Lia M Mo T Ludwig T Rajewsky K Dalla FR Transcription factor IRF4 controls plasma cell differentiation and class-switch recombination Nat Immunol 2006 7 773 782 10.1038/ni1357 16767092

[B12] Honda K Taniguchi T IRFs: master regulators of signalling by Toll-like receptors and cytosolic pattern-recognition receptors Nat Rev Immunol 2006 6 644 658 10.1038/nri1900 16932750

[B13] Dougherty DC Park HM Sanders MM Interferon regulatory factors (IRFs) repress transcription of the chicken ovalbumin gene Gene 2009 439 63 70 10.1016/j.gene.2009.03.016 19341784 PMC2749989

[B14] Sun BJ Chang MX Chen DL Nie P Gene structure and transcription of IRF-2 in the mandarin fish *Siniperca chuatsi *with the finding of alternative transcripts and microsatellite in the coding region Immunogenetics 2006 58 774 784 10.1007/s00251-006-0129-y 16871414

[B15] Sun BJ Chang MX Song Y Yao WJ Nie P Gene structure and transcription of IRF-1 and IRF-7 in the mandarin fish *Siniperca chuatsi* Vet Immunol Immunopathol 2007 116 26 36 10.1016/j.vetimm.2007.01.001 17289159

[B16] Zhang YB Hu CY Zhang J Huang GP Wei LH Zhang QY Gui JF Molecular cloning and characterization of crucian carp (*Carassius auratus *L.) interferon regulatory factor 7 Fish Shellfish Immunol 2003 15 453 466 10.1016/S1050-4648(03)00025-1 14550671

[B17] Yabu T Hirose H Hirono I Katagiri T Aoki T Yamamoto E Molecular cloning of a novel interferon regulatory factor in Japanese flounder, *Paralichthys olivaceus* Mol Mar Biol Biotechnol 1998 7 138 144 9628009

[B18] Holland JW Bird S Williamson B Woudstra C Mustafa A Wang T Zou J Blaney SC Collet B Secombes CJ Molecular characterization of IRF3 and IRF7 in rainbow trout, *Oncorhynchus mykiss*: functional analysis and transcriptional modulation Mol Immunol 2008 46 269 285 10.1016/j.molimm.2008.08.265 18805586

[B19] Stein C Caccamo M Laird G Leptin M Conservation and divergence of gene families encoding components of innate immune response systems in zebrafish Genome Biol 2007 8 R251 10.1186/gb-2007-8-11-r251 18039395 PMC2258186

[B20] Jiang Y Doolittle RF The evolution of vertebrate blood coagulation as viewed from a comparison of puffer fish and sea squirt genomes Proc Natl Acad Sci USA 2003 100 7527 7532 10.1073/pnas.0932632100 12808152 PMC164620

[B21] Cannon JP Haire RN Litman GW Identification of diversified genes that contain immunoglobulin-like variable regions in a protochordate Nat Immunol 2002 3 1200 1207 10.1038/ni849 12415263

[B22] Huang S Yuan S Guo L Yu Y Li J Wu T Liu T Yang M Wu K Liu H Genomic analysis of the immune gene repertoire of amphioxus reveals extraordinary innate complexity and diversity Genome Res 2008 18 1112 1126 10.1101/gr.069674.107 18562681 PMC2493400

[B23] Putnam NH Butts T Ferrier DEK Furlong RF Hellsten U Kawashima T Robinson-Rechavi M Shoguchi E Terry A Yu JK The amphioxus genome and the evolution of the chordate karyotype Nature 2008 453 1064 1071 10.1038/nature06967 18563158

[B24] Dehal P Satou Y Campbell RK Chapman J Degnan B Tomaso DA Davidson B Gregorio DA Gelpke M Goodstein DM The draft genome of *Ciona intestinalis*: insights into chordate and vertebrate origins Science 2002 298 2157 2167 10.1126/science.1080049 12481130

[B25] Nehyba J Hrdlickova R Bose HR Dynamic evolution of immune system regulators: the history of the interferon regulatory factor family Mol Biol Evol 2009 26 2539 2550 10.1093/molbev/msp167 19638535 PMC2767096

[B26] Wang W Yu H Long M Duplication-degeneration as a mechanism of gene fission and the origin of new genes in *Drosophila *species Nat Genet 2004 36 523 527 10.1038/ng1338 15064762

[B27] Babushok DV Ostertag EM Kazazian HH Jr Current topics in genome evolution: molecular mechanisms of new gene formation Cell Mol Life Sci 2007 64 542 554 10.1007/s00018-006-6453-4 17192808 PMC11138463

[B28] Zou J Chang M Nie P Secombes CJ Origin and evolution of the RIG-I like RNA helicase gene family BMC Evol Biol 2009 9 85 10.1186/1471-2148-9-85 19400936 PMC2686710

[B29] Li G Zhang J Sun Y Wang H Wang Y The evolutionarily dynamic IFN-inducible GTPase proteins play conserved immune functions in vertebrates and cephalochordates Mol Biol Evol 2009 26 1619 1630 10.1093/molbev/msp074 19369598

[B30] Qin BY Liu C Lam SS Srinath H Delston R Correia JJ Derynck R Lin K Crystal structure of IRF-3 reveals mechanism of autoinhibition and virus-induced phosphoactivation Nat Struct Biol 2003 10 913 921 10.1038/nsb1002 14555996

[B31] Kasahara M The 2R hypothesis: an update Curr Opin Immunol 2007 19 547 552 10.1016/j.coi.2007.07.009 17707623

[B32] Yan Postlethwait YL Gates MA Horne S Amores A Brownlie A Donovan A Egan ES Force A Gong Z Vertebrate genome evolution and the zebrafish gene map Nat Genet 1998 18 345 349 10.1038/ng0498-345 9537416

[B33] Amores A Force A Yan YL Joly L Amemiya C Fritz A Ho RK Langeland J Prince V Wang YL Zebrafish hox clusters and vertebrate genome evolution Science 1998 282 1711 1714 10.1126/science.282.5394.1711 9831563

[B34] Wotton KR Shimeld SM Comparative genomics of vertebrate Fox cluster loci BMC Genomics 2006 7 271 10.1186/1471-2164-7-271 17062144 PMC1634998

[B35] Eddy SR Profile hidden Markov models Bioinformatics (Oxford, England) 1998 14 755 763 10.1093/bioinformatics/14.9.755 9918945

[B36] Rice P Longden I Bleasby A EMBOSS: the European Molecular Biology Open Software Suite Trends Genet 2000 16 276 277 10.1016/S0168-9525(00)02024-2 10827456

[B37] Thompson JD Higgins DG Gibson TJ CLUSTAL W: improving the sensitivity of progressive multiple sequence alignment through sequence weighting, position-specific gap penalties and weight matrix choice Nucleic Acids Res 1994 22 4673 4680 10.1093/nar/22.22.4673 7984417 PMC308517

[B38] Tamura K Dudley J Nei M Kumar S MEGA4: Molecular Evolutionary Genetics Analysis (MEGA) software version 4.0 Mol Biol Evol 2007 24 1596 1599 10.1093/molbev/msm092 17488738

