# Supplementary material for: Global characterization of interferon regulatory factor (IRF) genes in vertebrates: Glimpse of the diversification in evolution
Source: BMC Immunol. 2010 May 5;11:22. doi: 10.1186/1471-2172-11-22 (PMC2885996; doi:10.1186/1471-2172-11-22)
Supplement: Additional file 1 — IRF genes identified in vertebrates. Table I IRF genes identified in vertebrates from fish to mammals, including human, mouse, dog, chicken, anole lizard, frog, zebrafish, stickleback, fugu, medaka, mandarin fish, rainbow trout, snakehead. [file 1471-2172-11-22-S1.PDF]

Table I

IRF genes identified in vertebrates from fish to mammals, including human, mouse, dog, chicken, anole lizard, frog, zebrafish, stickleback, fugu, medaka, mandarin fish, rainbow trout, snakehead.

IRF	Species	Chromosome / scaffold	Database ID	Closest human*	Identity / similarity
human IRF-1	*H. sapiens*	5	ENSG00000125347	-	-
human IRF-2	*H. sapiens*	4	ENSG00000168310	-	-
human IRF-3	*H. sapiens*	19	ENSG00000126456	-	-
human IRF-4	*H. sapiens*	6	ENSG00000137265	-	-
human IRF-5	*H. sapiens*	7	ENSG00000128604	-	-
human IRF-6	*H. sapiens*	1	ENSG00000117595	-	-
human IRF-7	*H. sapiens*	11	ENSG00000185507	-	-
human IRF-8	*H. sapiens*	16	ENSG00000140968	-	-
human IRF-9	*H. sapiens*	14	ENSG00000213928	-	-
mouse IRF-1	*M. musculus*	11	ENSMUSG00000018899	hsIRF-1	84.5% / 92.1%
mouse IRF-2	*M. musculus*	8	ENSMUSG00000031627	hsIRF-2	92.8% / 96.6%
mouse IRF-3	*M. musculus*	7	ENSMUSG00000003184	hsIRF-3	70.7% / 78.5%
mouse IRF-4	*M. musculus*	13	ENSMUSG00000021356	hsIRF-4	92.2% / 95.8%
mouse IRF-5	*M. musculus*	6	ENSMUSG00000029771	hsIRF-5	87.1% / 89.7%
mouse IRF-6	*M. musculus*	1	ENSMUSG00000026638	hsIRF-6	97.6% / 98.9%
mouse IRF-7	*M. musculus*	7	ENSMUSG00000025498	hsIRF-7	62.2% / 69.3%
mouse IRF-8	*M. musculus*	8	ENSMUSG00000041515	hsIRF-8	89.7% / 93.2%
mouse IRF-9	*M. musculus*	14	ENSMUSG00000002325	hsIRF-9	66.3% / 74.5%

dog IRF-1	*C. familiaris*	11	ENSCAFG00000000851	hsIRF-1	91.1% / 95.1%
dog IRF-2	*C. familiaris*	16	ENSCAFG00000007761	hsIRF-2	90.9% / 93.5%
dog IRF-3-seg	*C. familiaris*	1	ENSCAFG00000003594	hsIRF-3	65.9% / 72.9%
dog IRF-4-seg	*C. familiaris*	35	ENSCAFG00000009156	hsIRF-4	90.2% / 92.6%
dog IRF-5-seg	*C. familiaris*	14	ENSCAFG00000001611	hsIRF-5	80.0% / 81.7%
dog IRF-6	*C. familiaris*	7	ENSCAFG00000011971	hsIRF-6	85.3% / 85.6%
dog IRF-7-seg	*C. familiaris*	18	ENSCAFG00000006557	hsIRF-7	37.4% / 41.0%
dog IRF-8	*C. familiaris*	5	ENSCAFG00000019919	hsIRF-8	91.8% / 94.8%
dog IRF-9	*C. familiaris*	8	ENSCAFG00000012007	hsIRF-9	73.4% / 80.7%
dog IRF-10-seg	*C. familiaris*	24	ENSCAFG00000007099	hsIRF-4	38.1% / 49.2%
chicken IRF-1	*G. gallus*	13	ENSGALG00000006785	hsIRF-1	60.3% / 76.1%
chicken IRF-2	*G. gallus*	4	ENSGALG00000010642	hsIRF-2	83.5% / 92.0%
chicken IRF-4	*G. gallus*	2	ENSGALG00000012830	hsIRF-4	84.3% / 92.0%
chicken IRF-5-seg	*G. gallus*	Un_random:19690742:19694139	De novo predicted	hsIRF-5	53.3% / 65.3%
chicken IRF-6	*G. gallus*	26	ENSGALG00000001405	hsIRF-6	76.9% / 82.6%
chicken IRF-7	*G. gallus*	5	ENSGALG00000014297	hsIRF-7	39.0% / 49.7%
chicken IRF-8	*G. gallus*	11	ENSGALG00000005757	hsIRF-8	73.6% / 84.6%
chicken IRF-10-seg	*G. gallus*	20	ENSGALG00000006448	hsIRF-4	43.0% / 55.5%
anole lizard IRF-1	*A. arolinensis*	Scaf_1	ENSACAG00000012699	hsIRF-1	64.6% / 50.9%
anole lizard IRF-2	*A. arolinensis*	Scaf_308	ENSACAG00000002786	hsIRF-2	86.1% / 76.8%
anole lizard IRF-3	*A. arolinensis*	Scaf_270	ENSACAG00000014028	hsIRF-3	45.6% / 32.7%
anole lizard IRF-4-seg	*A. arolinensis*	Scaf_243	ENSACAG00000017711	hsIRF-4	86.9% / 79.0%
anole lizard IRF-5-seg	*A. arolinensis*	Scaf_7087	ENSACAG00000008511	hsIRF-5	30.6% / 27.0%
anole lizard IRF-6	*A. arolinensis*	Scaf_13	ENSACAG00000005931	hsIRF-6	89.4% / 82.9%
anole lizard IRF-7	*A. arolinensis*	Scaf_73	ENSACAT00000015053	hsIRF-7	40.7% / 31.4%

anole lizard IRF-8	*A. arolinensis*	Scaf_781	ENSACAG00000005409	hsIRF-8	79.0% / 68.0%
anole lizard IRF-9	*A. arolinensis*	Scaf_474	ENSACAG00000003946	hsIRF-9	49.9% / 37.5%
anole lizard IRF-10-seg	*A. arolinensis*	Scaf_1561	ENSACAG00000004016	hsIRF-8	28.0% /38.9%
frog IRF-1	*X. tropicalis*	Scaf_93	ENSXETG00000016586	hsIRF-1	54.4% / 68.6%
frog IRF-2	*X. tropicalis*	Scaf_90	ENSXETG00000001536	hsIRF-2	67.5% / 78.2%
frog IRF-3	*X. tropicalis*	Scaf_587	ENSXETG00000002863	hsIRF-3	32.0% / 48.5%
frog IRF-4-seg	*X. tropicalis*	Scaf_211	ENSXETG00000011413	hsIRF-4	56.1% / 64.8%
frog IRF-5	*X. tropicalis*	Scaf_11	ENSXETG00000015005	hsIRF-9	26.4% / 35.7%
frog IRF-6-1	*X. tropicalis*	Scaf_375:785262-798486	ENSXETG00000018674	hsIRF-6	77.3% / 85.8%
frog IRF-6-2	*X. tropicalis*	Scaf_375:760016-772977	ENSXETG00000018661	hsIRF-6	77.3% / 85.8%
frog IRF-7-seg	*X. tropicalis*	Scaf_398	ENSXETG00000006030	hsIRF-7	30.8% / 43.9%
frog IRF-8-seg	*X. tropicalis*	Scaf_120	ENSXETG00000003561	hsIRF-8	53.2% / 65.3%
frog IRF-9-seg	*X. tropicalis*	Scaf_439: 493050 - 508689	De novo predicted	hsIRF-9	36.7% / 52.2%
frog IRF-10-seg	*X. tropicalis*	Scaf_1295	ENSXETG00000002549	hsIRF-4	35.2% / 49.1%
fugu IRF-1	*T. rubripes*	Scaf_71	ENSTRUG00000004321	hsIRF-1	37.8% / 53.0%
fugu IRF-2	*T. rubripes*	Scaf_410	ENSTRUG00000001842	hsIRF-2	47.7% / 59.9%
fugu IRF-3	*T. rubripes*	Scaf_3	ENSTRUG00000012031	hsIRF-3	29.0% / 41.5%
fugu IRF-4-1	*T rubripes*	Scaf_351	ENSTRUG00000002946	hsIRF-4	52.5% / 63.3%
fugu IRF-4-2	*T. rubripes*	Scaf_107	ENSTRUG00000011568	hsIRF-4	49.7% / 57.2%
fugu IRF-5	*T. rubripes*	Scaf_626	ENSTRUG00000000641	hsIRF-6	33.4% / 42.0%
fugu IRF-6	*T. rubripes*	Scaf_87	ENSTRUG00000006951	hsIRF-6	59.9% / 70.4%
fugu IRF-7	*T. rubripes*	Scaf_119	ENSTRUG00000011418	hsIRF-7	27.7% / 41.0%
fugu IRF-8	*T. rubripes*	Scaf_14	ENSTRUG00000018213	hsIRF-8	31.8% / 39.8%
fugu IRF-9	*T. rubripes*	Scaf_96	ENSTRUG00000017722	hsIRF-9	31.1% / 45.9%
fugu IRF-10	*T. rubripes*	Scaf_180	ENSTRUG00000002917	hsIRF-4	33.3% / 43.6%

fugu IRF-11-seg	*T. rubripes*	Scaf_265	ENSTRUG00000000250	hsIRF-1	21.8% /30.1%
zebrafish IRF-1	*D. rerio*	21	ENSDARG00000032768	hsIRF-1	43.5% / 55.9%
zebrafish IRF-2-seg	*D. rerio*	1	ENSDARG00000007387	hsIRF-2	44.6% / 57.0%
zebrafish IRF-3	*D. rerio*	12	ENSDARG00000076251	hsIRF-3	24.9% / 35.1%
zebrafish IRF-4-c2	*D. rerio*	2	ENSDARG00000006560	hsIRF-4	60.7% / 73.9%
zebrafish IRF-4-c20	*D. rerio*	20	ENSDARG00000055374	hsIRF-4	52.0% / 64.7%
zebrafish IRF-4-scafNA1075-seg	*D. rerio*	Scaf_NA1075	ENSDARG00000035766	hsIRF-4	36.3% / 47.4%
zebrafish IRF-5	*D. rerio*	4	ENSDARG00000045681	hsIRF-5	43.5% / 51.8%
zebrafish IRF-6	*D. rerio*	22	ENSDARG00000043296	hsIRF-6	63.5% / 73.9%
zebrafish IRF-7	*D. rerio*	25	ENSDARG00000045661	hsIRF-7	26.3% / 38.5%
zebrafish IRF-8	*D. rerio*	18	ENSDARG00000056407	hsIRF-8	55.4% / 70.9%
zebrafish IRF-9	*D. rerio*	12	ENSDARG00000016457	hsIRF-9	28.7% / 40.8%
zebrafish IRF-10	*D. rerio*	23	ENSDARG00000027658	hsIRF-8	39.0% / 52.7%
zebrafish IRF-11	*D. rerio*	13	ENSDARG00000043492	hsIRF-1	31.1% / 44.4%
stickleback IRF-1	*G. aculeatus*	groupVII	ENSGACG00000020876	hsIRF-1	35.8% / 51.0%
stickleback IRF-2-seg	*G. aculeatus*	groupIV	ENSGACG00000018763	hsIRF-2	51.6% / 63.7%
stickleback IRF-3	*G. aculeatus*	groupV	ENSGACG00000007945	hsIRF-3	29.3% / 44.2%
stickleback IRF-4-1	*G. aculeatus*	groupIII	ENSGACG00000016461	hsIRF-4	56.8% / 67.7%
stickleback IRF-4-2	*G. aculeatus*	groupVIII	ENSGACG00000004966	hsIRF-4	52.7% / 63.1%
stickleback IRF-5	*G. aculeatus*	Scaf_90	ENSGACG00000000695	hsIRF-5	50.0% / 61.0%
stickleback IRF-6-seg	*G. aculeatus*	Scaf_27	ENSGACG00000000990	hsIRF-6	56.0% / 66.3%
stickleback IRF-7-seg	*G. aculeatus*	groupXIX	ENSGACG00000011372	hsIRF-7	28.9% / 44.6%
stickleback IRF-8	*G. aculeatus*	groupII	ENSGACG00000015958	hsIRF-8	50.1% / 62.9%
stickleback IRF-9-seg	*G. aculeatus*	Scaf_213	ENSGACG00000001697	hsIRF-9	29.5% / 42.8%
stickleback IRF-10-seg	*G. aculeatus*	Scaf_68	ENSGACG00000015123	hsIRF-4	37.2% / 47.8%

stickleback IRF-11-seg	*G. aculeatus*	groupII	ENSGACG00000016299	hsIRF-1	23.3% / 29.8%
medaka IRF-1	*O. latipes*	14	ENSORLG00000001131	hsIRF-1	38.1% / 50.9%
medaka IRF-2	*O. latipes*	10	ENSORLG00000008574	hsIRF-2	42.8% / 49.6%
medaka IRF-3	*O. latipes*	19	ENSORLG00000006703	hsIRF-3	30.4% / 48.8%
medaka IRF-4-1	*O. latipes*	4	ENSORLG00000012712	hsIRF-4	44.7% / 52.5%
medaka IRF-4-2	*O. latipes*	17	ENSORLG00000017242	hsIRF-4	56.7% / 68.5%
medaka IRF-5	*O. latipes*	Scaf_1365	ENSORLG00000020118	hsIRF-5	51.0% / 61.5%
medaka IRF-6	*O. latipes*	5	ENSORLG00000000511	hsIRF-6	60.6% / 71.2%
medaka IRF-8	*O. latipes*	22	ENSORLG00000018216	hsIRF-8	49.0% / 64.0%
medaka IRF-9	*O. latipes*	20	ENSORLG00000013335	hsIRF-9	34.1% / 48.0%
medaka IRF-10	*O. latipes*	7	ENSORLG00000011745	hsIRF-9	34.0% / 48.9%
medaka IRF-11-seg	*O. latipes*	3	ENSORLG00000011716	hsIRF-1	22.4% / 31.0%
mandarin fish IRF-1	*S. chuatsi*	-	GI:55775523	hsIRF-1	38.9% / 54.9%
mandarin fish IRF-2	*S. chuatsi*	-	GI:45775308	hsIRF-2	45.4% / 55.2%
mandarin fish IRF-7	*S. chuatsi*	-	GI:55775529	hsIRF-7	29.4% / 43.9%
rainbow trout IRF-1	*O. mykiss*	-	GI:21886658	hsIRF-1	36.3% / 50.9%
rainbow trout IRF-2	*O. mykiss*	-	GI:15072469	hsIRF-2	53.3% / 64.4%
snakehead IRF-1	*C. argus*	-	GI:118140104	hsIRF-1	39.6% / 55.7%
snakehead IRF-2a	*C. argus*	-	GI:118140106	hsIRF-2	52.9% / 67.2%
snakehead IRF-2b	*C. argus*	-	GI:125487131	hsIRF-2	51.3% / 66.4%
snakehead IRF-7	*C. argus*	-	GI:118140102	hsIRF-7	29.2% / 43.6%

*indicating closest human counterparts.
