# Supplementary material for: Global characterization of interferon regulatory factor (IRF) genes in vertebrates: Glimpse of the diversification in evolution
Source: BMC Immunol. 2010 May 5;11:22. doi: 10.1186/1471-2172-11-22 (PMC2885996; doi:10.1186/1471-2172-11-22)
Supplement: Additional file 2 — Homologues of IRFs in non-vertebrate deuterostomes. Table II Sequence information of homologues of IRFs in non-vertebrate deuterostomes including sea squirt, lancelet, sea urchin and acorn worm. [file 1471-2172-11-22-S2.PDF]

Table II

Sequence information of homologues of IRFs in non-vertebrate deuterostomes including sea squirt, lancelet, sea urchin and acorn worm.

IRF-like	Species	Chromosome/ scaffold	Database ID	Closest human*	Identity / similarity	Equivalent name*
sea squirt IRF-like-scaf_162	*C. intestinalis*	Scaf_162	ENSCINP00000001474	hsIRF-4	23.7% / 36.1%	CI3
sea squirt IRF-like-3q-1	*C. intestinalis*	3q:2590577-2595970	ENSCING00000012684	hsIRF-6	19.6% / 31.4%	CI5
sea squirt IRF-like-3q-2	*C. intestinalis*	3q:4167392-4170835	ENSCING00000008908	hsIRF-3	21.0% / 35.0%	CI7
sea squirt IRF-like-3q-3	*C. intestinalis*	3q:4171362-4175135	ENSCING00000008909	hsIRF-4	20.0% / 37.0%	CI8
sea squirt IRF-like-12q	*C. intestinalis*	12q	ENSCING00000002820	hsIRF-5	23.4% / 38.2%	CI4
sea squirt IRF-like-14p	*C. intestinalis*	14p	ENSCING00000005877	hsIRF-6	19.6% / 31.4%	CI2
sea squirt IRF-like-seg-1	*C. intestinalis*	NW_0019548621	De novo predicted	hsIRF-9	18.2% / 31.8%	CI9
sea squirt IRF-like-seg-2	*C. intestinalis*	Scaf_6701	De novo predicted	hsIRF-1	5.4% / 9.0%	CI6
sea squirt IRF-like-seg-3	*C. intestinalis*	AABS01000045.1	De novo predicted	hsIRF--1	18.7% / 24.7%	CI1
lancelet IRF-like-scaf_187/1-seg	*B. floridae*	Scaf_187: 1243114-1261719	Gi: 260798024	hsIRF-8	26.3% / 35.8%	BF3
lancelet IRF-like-scaf_172	*B. floridae*	Scaf_172	Gi: 260801909	hsIRF-7	19.4% / 27.4%	BF5
lancelet IRF-like-scaf_12	*B. floridae*	Scaf_12	Gi:260835395	hsIRF-1	22.7% / 33.4%	BF2
lancelet IRFlike-scaf_196	*B. floridae*	Scaf_196:4150376-4155105	De novo predicted	hsIRF-4	21.5% / 34.5%	BF1
lancelet IRFlike-scaf_136	*B. floridae*	Scaf_136:3504336-3539227	De novo predicted	hsIRF-1	18.9% / 29.9%	BF6
lancelet IRF-like-scaf_48	*B. floridae*	Scaf_48	Gi:260824565	hsIRF-4	20.0% / 30.5%	BF11
lancelet IRF-like-scaf_187/2	*B. floridae*	Scaf_187: 1269355-1275015	Gi:260798026	hsIRF-8	27.7% / 43.6%	BF9
lancelet IRF-like-scaf_27	*B. floridae*	Scaf_27	Gi:260830756	hsIRF-8	16.4% / 25.5%	BF4
lancelet IRF-like-scaf_7	*B. floridae*	Scaf_7	Gi:260836206	hsIRF-5	26.1% / 41.7%	BF10
lancelet IRFlike-scaf_187/3	*B. floridae*	Scaf_187: 1276857-1282310:	Gi:260798028	hsIRF-1	18.0% / 28.6%	BF8
sea urchin IRF-like-1	*S. purpuratus*	NW_001318745.1	Gi:115730749	hsIRF-1	22.8% / 34.6%	SP1

sea urchin IRFlike-2	*S. purpuratus*	NW_001304107.1	Gi:72007640	hsIRF-5	20.7% / 32.7%	SP2
acorn worm IRF-like-1	*S. kowalevskii*	ACQM01059771.1	De novo predicted	hsIRF-2	27.0% / 37.1%	SK1
acorn worm IRF-like-2	*S. kowalevskii*	ACQM01005341.1	De novo predicted	hsIRF-8	24.2% / 36.0%	SK2
acorn worm IRF-like-3-seg	*S. kowalevskii*	ACQM01130185.1	De novo predicted	hsIRF-1	10.8% / 17.4%	SK3

*indicating alternative but equivalent gene symbols in Nehyba *et al*. (2009)
